# Characterization of algal community composition and structure from the nearshore environment, Lake Tahoe (United States)

**DOI:** 10.3389/fevo.2022.1053499

**Published:** 2023-01-20

**Authors:** Paula J. Noble, Carina Seitz, Sylvia S. Lee, Kalina M. Manoylov, Sudeep Chandra

**Affiliations:** 1 Department of Geological Sciences and Engineering and Global Water Center, University of Nevada Reno, Reno, NV, United States; 2 Centro Regional Universitario Bariloche (CRUB)-CONICET, Departamento de geologia y petroleo, Universidad Nacional del Comahue, San Carlos de Bariloche, Argentina; 3 Office of Research and Development, U.S. Environmental Protection Agency, Washington, DC, United States; 4 Department of Biological and Environmental Sciences, Georgia College & State University, Milledgeville, GA, United States; 5 Department of Biology and Global Water Center, University of Nevada Reno, Reno, NV, United States

**Keywords:** diatom, voucher flora, lake Tahoe, periphyton, cyanobacteria

## Abstract

Periphyton assemblages from the nearshore environment of the west (California) side of Lake Tahoe, were analyzed to determine their taxonomic composition and community structure across habitats and seasons. Lake Tahoe is the second deepest lake in the US and an iconic oligotrophic subalpine lake with remarkable transparency. It has experienced offshore cultural eutrophication since the 1960s with observations of nuisance nearshore algal growth since the mid 2000s attributed to anthropogenic stressors. Samplings from November 2019–September 2020 provide useful snapshots against which older monitoring may be contextualized. A voucher flora, complete with descriptions, photo-documentation and referencing to species concepts employed, was created as a method of providing reproducible identification and enumeration of algal species, and more seamless reconciliation of detailed taxonomic data with future monitoring projects. The eulittoral zone (0–2 m) is seasonally dominated by elongate araphid (*Synedra, Ulnaria*) and stalked or entubed diatoms (*Gomphonema, Cymbella, Encyonema*). The sublittoral zone (>2 m) is dominated by a nitrogen-fixing *Epithemia*-cyanobacteria assemblage with less seasonal changes in dominance and composition that expanded to impinge on the 2 m depths of the eulittoral zone in the Fall. Sublittoral epipsammic samples, despite their proximity to rocks, had a very distinct diatom composition and high species dominance, similar to what was seen in the Fall eulittoral samples, with high numbers of *Staurosirella* chains and small biraphid diatoms. The deeper samples at 30 and 50 m contained high numbers of live *Epithemia*, and indicate a thriving sublittoral assemblage at these greater depths, but with less biomass. The 2019–20 data show many of the same diatom taxa observed in the 1970’s and 1980’s but with changes in species dominance. Notably, there was less of the green alga *Mougeotia*, when compared to the 1970’s data, and a higher dominance by nitrogen fixing *Epithemia* in the sublittoral zone, persisting year-round. These new data show roughly double the algal species biodiversity that had been documented previously in the Lake Tahoe nearshore, and is largely attributed to the methods employed. Adopting these new methods in future monitoring efforts should improve harmonization of taxonomic data and help advance our knowledge of the contributions to nearshore cultural eutrophication.

## 1. Introduction

Known for its remarkable water clarity, Lake Tahoe is an iconic subalpine oligotrophic lake located in the Sierra Nevada at an elevation of 1900 m.a.s.l., straddling the state line between California and Nevada. It is a large, steep-sided deep lake (maximum depth of 501 m) with a littoral zone encompassing ~19% of the lake’s total surface area (Goldman, 1974). Progressive, offshore cultural eutrophication has been documented since the 1960’s with a reduction in clarity by 10 m, and has resulted in changes to the lake benthos including alterations to the amount of bottom algal production (Chandra et al., 2005). In recent decades, local residents have observed nearshore, nuisance algal blooms that has been attributable to the introduction of species, changes in the timing of groundwater flow, and warmer temperatures (Naranjo et al., 2019; Vadeboncoeur et al., 2021). Since the 1970’s a large body of research has characterized many aspects of the limnology and investigated causes for water quality decline (TERC, 2022), with some research suggesting little or decreasing change in eulittoral biomass (Atkins et al., 2021). Few published studies address species-level algal biodiversity and/or taxonomy in Lake Tahoe, especially with respect to the periphyton. Herein, the term periphyton, ingrained in the Tahoe literature, simply refers to benthic algae.

Species-level enumeration of periphyton, especially diatom taxa from lakes and rivers, is an important component of water quality monitoring. Documenting project-specific morphological species concepts enables harmonization with existing and future monitoring data, and is vital to maintain informative long-term records and maximize data use (Potapova et al., 2022). Given the high species diversity and degree of endemism in freshwater diatom communities, as well as rapid developments in freshwater diatom taxonomy, there are no single taxonomic references that may be satisfactorily employed for species-level identification. Thus, the development of localized taxonomic references becomes essential in documenting the biodiversity and community structure. In Lake Tahoe, taxonomic surveys of periphyton were conducted in the 1960’s through 1980’s (Goldman, 1974; Goldman and De Amezaga, 1975; Van Landingham, 1987), and investigations of community composition of the periphyton in eulittoral and sublittoral zones additionally provide limited composition data (Aloi et al., 1988; Atkins et al., 2021). These resources contain only species lists, lacking photo-documentation, reference citation information, specimen accession numbers, and species notes affirming the taxonomic concepts that were employed. Taxonomically based work is sparse for Tahoe diatom periphyton, limited to select species of gomphonemoids (Kociolek and Stoermer, 1988), and as such the earlier species lists are difficult to harmonize with modern taxonomic usage.

In the context of a project with support from the California Lahontan Water Quality Control Board that serves to examine factors effecting nuisance algal growth in the nearshore, a diatom voucher flora was constructed to serve as the taxonomic identification resource for the west shore of Lake Tahoe. Voucher floras, as discussed by Bishop et al. (2017) and Alers-García et al. (2021), serve as working laboratory documents that can provide taxonomic consistency and quality control for algal enumeration during a project lifetime. They are devised as in-house taxonomic references for a specific project that provides a consistent method for analyzing large sample sets that often employ multiple analysts (Tyree et al., 2020a), and may be conducted over an extended period of years. Voucher floras differ from traditional monographic descriptions of diatom floras in that they document both well-established species, and also unidentified taxa whose taxonomic affinity to published scientific names is unresolved for a variety of reasons. Herein, we provide a diatom voucher flora and apply this method to enumeration of seasonal data from epilithic (=rock substrate) and epipsammic (=sand substrate) habitats in order to best characterize the community composition and structure. We additionally use soft algal community analysis to determine relative abundances of all algal groups in the nearshore environment from 0.5 to 50 m depth, including live diatoms, through observation of physiologically active chloroplasts within diatom frustules. The taxonomic survey and identification reference appended in the Supplementary material can serve as a foundation for future biodiversity work in the Lake Tahoe basin, and provide support for understanding why the nearshore environment in pristine lakes are undergoing enhanced algal growth and degrading water quality conditions.

## 2. Materials and methods

**FIGURE 1:**
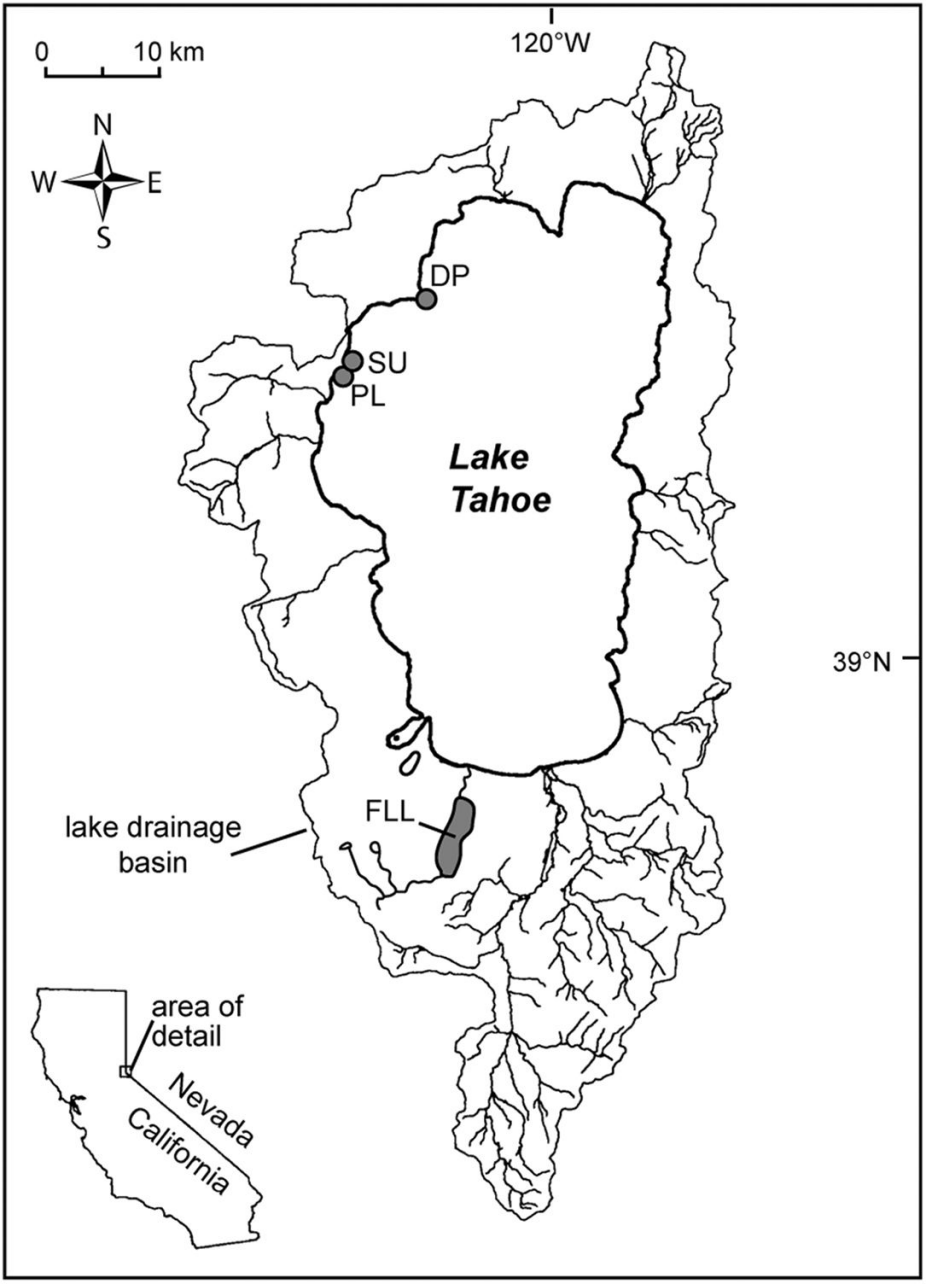
Locality map showing the extent of the watershed for Lake Tahoe, major drainages, and the 3 sampling localities on the west shore. SU: Sunnyside Harbor; PL: Pineland, and DP: Dollar Point, FLL: Fallen Leaf Lake.

The diatom voucher flora constructed for the Lake Tahoe nearshore area contains images of the taxa encountered in the examination of 29 samples collected from rock scrapings and surface sediment from 0.5 m to 8 m water depth in the vicinity of Sunnyside Harbor, Pineland, and Dollar Point from April 2019 through September 2020 (Figure 1; Table 1) as part of an evaluation of nuisance algal growth in this area of the lake, conducted for California’s Lahonton Regional Water Quality Control Board. Both the eulittoral zone, a high energy wave zone 0-2 m depth, and the sublittoral zone (>2 m) were sampled because previous work has shown distinctions in algal assemblages (Loeb and Reuter, 1984; Aloi et al., 1988; Atkins et al., 2021). A subset of 19 samples were then enumerated to determine relative species abundance. The enumerated samples include 8 samples from rock scrapes in the eulittoral zone in Spring (4-23-19), early Summer (6-1-20), and Fall (9-29-20) seasons spanning the 3 sites, 8 samples from rock scrapings in the sublittoral zone, and 3 samples from surface sediment scrapings in the sublittoral zone. All sublittoral zone samples are from outside of Sunnyside Harbor at approximately 8 m depth from Fall (November 12 and 13, 2019) and Spring (July 21, 2020) seasons. Enumerated data were used as a means of evaluating community composition and seasonal differences in epilithic eulittoral and sublittoral zones, and the epipsammic community.

The 29 diatom samples were processed using a hydrogen peroxide digestion method, where samples were heated for ~1 h in a 15% solution of peroxide. Samples were decanted then acidified using 20% HCl solution, heated for an additional hour, then decanted multiple times over the course of a week to remove acid. Cleaned siliceous slurries were then pipetted onto glass coverslips and permanent mounts of strewn slides were made using Zrax mounting medium. Slides were scanned and species documented with transmitted light microscopy, using DIC, and a 100x oil immersion lens on an Olympus BX51 microscope. Photomicrographs were taken using a Leica DMLB II microscope and Leica DMC6200 camera (Leica Microsystems, Wetzlar, Germany) and then sorted into operational taxonomic units (OTU) that were each assigned a provisional species code. In cases where the genus assignment of an OTU appeared clear, the species code used was a combination of the abbreviated genus name and a numerical designation (e.g., NAV01 for a species of *Navicula*). In some cases, taxa were later assigned to a different genus after the OTU had been in use (e.g., ENC04 = *Cymbella neoleptoceros*; Krammer, 2002). In cases where genus assignment would require investigation into the literature, the species code was abbreviated from a broader morphologic group (e.g., FRAG01 for a species of fragilarioid). Following the initial designation of OTUs, identification of diatom OTUs to the species level was undertaken using a variety of resources available, including the Diatoms of North America website (Spaulding et al., 2021), and many other resources from both North America and Europe (see Supplementary material). Initial identifications underwent a reconciliation process, to cross-check identifications and split out additional OTUs from morphologically similar arrays. Additionally, the voucher flora was harmonized with an unpublished flora constructed as part of a MS thesis by Stratton (2013) on Fallen Leaf Lake, a smaller subalpine lake in the Tahoe watershed (Figure 1). The Fallen Leaf Lake flora has served as an internal reference in author Noble’s laboratory, that is used for enumeration of Holocene Lake cores in the Tahoe basin (Ball et al., 2018; Johnson et al., 2018) and shares many taxa with the present sample set. All slides examined in this study have been accessioned in the Natural History Museum at the University of Nevada Reno and thus are available to the public and other scientists for examination.

Samples were then enumerated by counting n = 200 valves across multiple transects of the strewn slides. For broken valves, those greater than half were counted. All data are reported as percentage valve counts and not converted to biovolume data, as is typical in many of the Tahoe enumeration work done by Utermöhl chambers and inverted light microscopy (i.e., Goldman, 1974). The enumerated data were analyzed and plotted using the software PAST v.3.24 (Hammer et al., 2001) to look for differences between epipsammic versus epilithic habitats and seasonal variations, using Principal Components Analysis (PCA) and indices of species richness and species dominance. The PCA analysis was used to determine the number of statistically significant axes (Birks, 2012).

In addition to diatom enumeration, a suite of 13 samples was separately collected from transects at Sunnyside Harbor from 0.5 to 50 m water depth for algal identification and enumeration to better characterize the entire periphyton community, especially the soft-bodied algae. Samples included 7 sediment and rock scrapes from the eulittoral zone from 3 seasons (3/9/20, 6/1/20, and 9/29/20), 2 rock scrapes at 8 m, and an additional 4 rock and sediment scrapes going down to 50 m (Table 2). Samples were collected and preserved in Lugol’s solution (APHA, 2012). Lugol’s preserved samples can be stored for several years. With Lugol’s preservation, shape, size, colony formation, structures, etc. are useful for identification, yet it prevents any taxonomic characteristics of color since starch in a sample is turned black due to the iodine. These samples were then examined at 400x magnification, and a minimum of 300 algal natural units were enumerated including live diatoms using a Palmer-Maloney cell (Palmer and Maloney, 1954) with ceramic chamber with a depth of 0.4 mm holding a volume of 0.1 ml. Counts were performed on a Leica DM2500 microscope with a DFC295 Camera (Leica Microsystems, Wetzlar, Germany). A minimum of 300 units was chosen, compared to the 200 valve counts for the diatom enumerations on the permanent mounts, in order to capture more of the soft-bodied assemblage.

Lugol’s samples were shaken to homogenize, and checked for unit density not to exceed 25 units per field of view of the counting chamber. Number of cells within each colony unit were estimated. For diatoms only frustules with a visible, healthy looking chloroplast were enumerated. Dead diatoms were enumerated additionally and allowed for the determination of the proportion of live to dead diatoms. A natural algal unit is defined as a natural grouping of algae (e.g., a unicellular flagellate or isolated cell; an individual filament; or a connected cluster or colony). Unlike the soft-bodied algal counting units, each diatom frustule is always a natural counting unit, even if attached to other frustules in a chain. The main purpose of using ‘natural counting units’ is to prevent cells from large colonial or filamentous forms from dominating a count. That also allows documentation of higher diversity with just 300 units enumerated. After counting, a minimum of 10 fields of view were scanned for each sample, or until there was no new taxa observed in five fields of view. In samples where coccoid colonies were in high abundance, and Lugol’s preservation (which can obscure pigmentation color) did not allow recognition of cyanobacteria versus chlorophyte colonies, the algal material was emptied in a small (2.5 cm diameter) Petri dish and diluted with distilled water to check for color and finalize the identification. The soft-bodied taxa were assigned OTUs and names using a variety of references, including Stancheva et al. (2014), Komárek and Hauer (2015), and Wehr et al. (2015). Taxa were harmonized to the names used in the SWAMP (Surface Water Ambient Monitoring Program) database used by the California Water Boards (Table 3). Taxon specific biomass was calculated based on cell number times cell biovolume. Biovolume estimations were based on measurements of the dimensions of a minimum of 10 cells, including their length, width, height, and diameter (depending on the shape, Hillebrand et al., 1999) of common taxa (i.e., wih a relative abundance higher than 10 units). Taxon biomass was determined by multiplying cell density and cell biovolume (μm^3^).

## 3. Results

### 3.1. Community composition

Counts of the total assemblage from Lugol’s preservation show most samples were dominated by diatoms, followed by filamentous and coccoid forms of cyanobacteria, and lesser amounts of green algae (Table 3). A few representatives of Dinoflagellata and Euglenophyta were also documented. Many filamentous and colonial algae were fragmented from collection processes and differentiation of genera like *Calothrix, Dichothrix, Dasygloea* etc. was inconclusive. Diatoms comprised over 90% of the biovolume and cell counts, regardless of depth, habitat or season. In terms of biomass, algae were relatively sparse in the 0.5 m epilithic samples, comprising a thin biofilm, but increased in samples greater than 2 m depth. Dead diatom frustule accumulations were highest in the sediment samples regardless of season, and increased in the deeper samples in the transect. Live *Epithemia* and *Staurosirella* chains were observed in all sublittoral samples, including the deepest samples, and comprised nearly 100% of the live algal biovolume at 30 and 50 m depth.

The observed OTU diversity consisted of 115 diatom species spanning 44 genera, as identified in the cleaned strewn slides, and 78 soft bodied taxa observed in the Lugol’s preparations. Of the diatom taxa, 87 are formally described species or varieties of species known in the published diatom literature, an additional 28 were left in open nomenclature with only a provisional species code designated for this project. Given that this was a benthic survey, many of the diatoms documented were benthic stalked and prostrate forms. A small percentage of phytoplankton were included, and their presence was attributed to their entrainment in algal mats, or adjacent sediment. Also present were a number of attached lotic forms which may represent washed in species entering from Ward Creek, and smaller inflows nearby.

**FIGURE 2:**
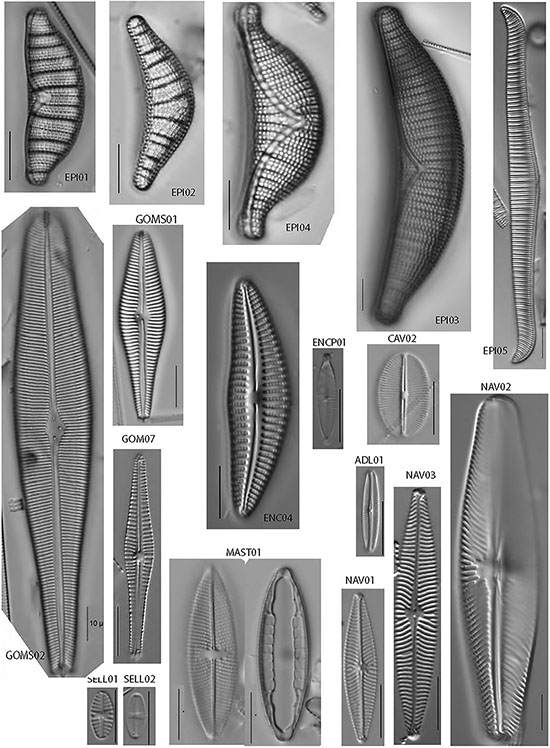
Light micrographs of common epithemioid and biraphid diatoms identified in this study. All scale bars are 10 microns in length.

**FIGURE 3:**
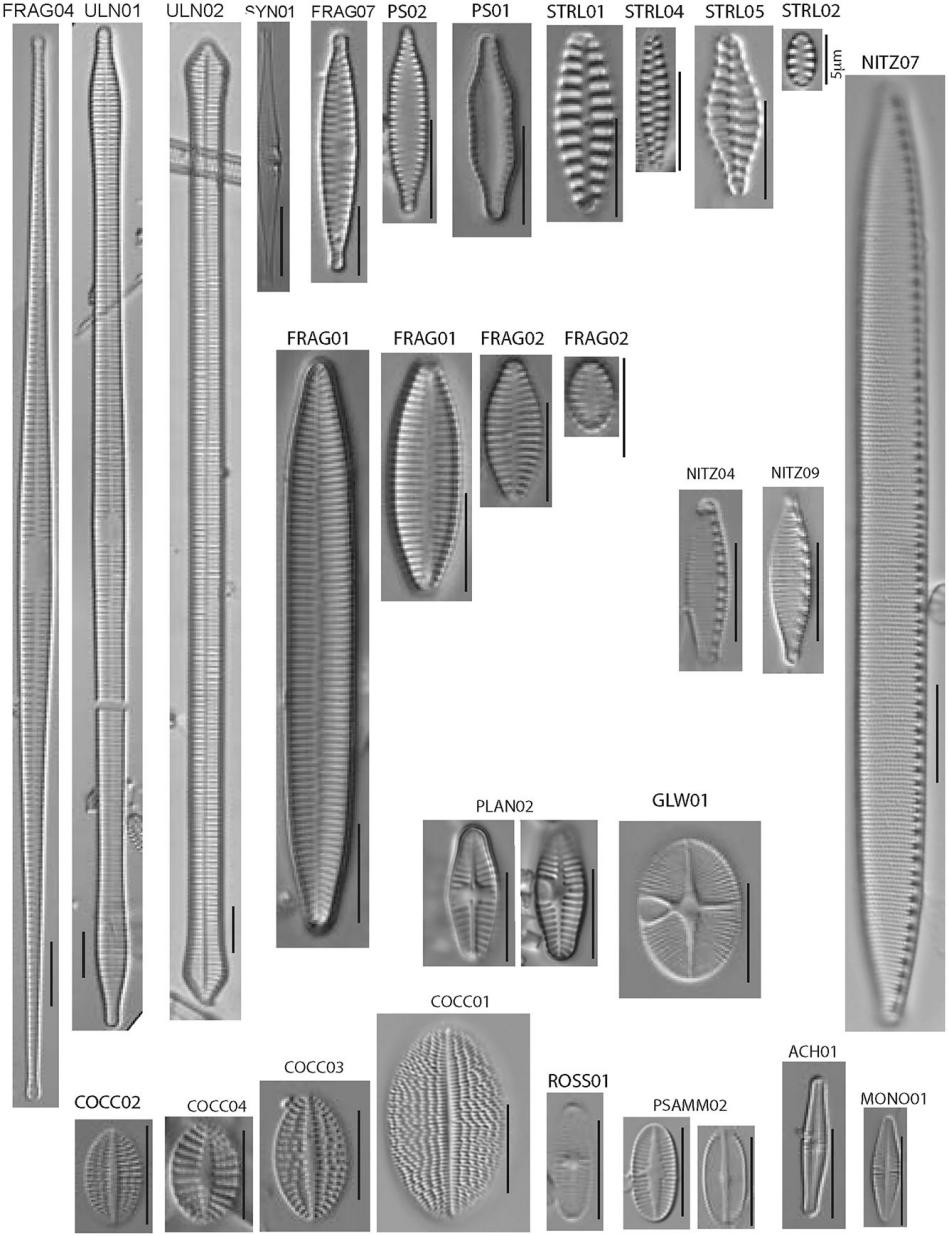
Transmitted light photomicrographs of common araphid, nitzschioid, and monoraphid diatoms identified in this study. All scale bars are 10 microns in length unless otherwise noted.

The most common members of the diatom assemblage in the samples included fragilarioids and stalked or entubed asymmetrical biraphids. The fragilarioids were dominated by species assigned to *Fragilaria*, *Synedra, Staurosirella, Staurosira*, *Pseudostaurosira*, and *Ulnaria*. The stalked or entubed biraphids were abundant and dominated by species of *Epithemia*, *Encyonema, Gomphonema*, and *Gomphoneis*, and to a lesser extent by species of *Amphora, Cymbopleura*, *Gomphosphenia*, and *Reimeria*. Large solitary biraphid forms were a consistent component of the flora and include several species of *Navicula* and *Nitzschia*. Smaller biraphids also made up a notable component, the common species being assigned to *Adlafia*, *Mastogloia, Encyonopsis, Sellaphora, Geissleria, Cavinula*, and *Brachysira*. A smaller component of the flora consisted of monoraphids, the most common of which belong to the genera *Psammothidium, Cocconeis, Gliwiczia*, and *Planothidium*. The 37 most common taxa from the enumerations appear in Table 4, along with abundance and ecological notes, and images of the most common taxa appear in Figures 2, 3.

Additional reference material for the taxonomic identifications and enumerations is provided as Supplementary material. Supplementary Table 1 is the diatom voucher file; a complete list of the taxa, their basionym, and references used in making taxonomic identifications for each species. Also in Supplementary Table 1 are cross-referencing to identifications in the Fallen Leaf Lake voucher flora of Stratton (2013), and the taxonomic list of periphyton from Goldman (1974). Diatom species counts are found in Supplementary Table 2, and the whole floral counts from Lugol’s preserved samples appear in Supplementary Table 3. Supplementary Data Sheet 1 contains photomicrographs of the common soft-bodied algae observed. Supplementary Data Sheet 2 contains photomicrographs of all diatom taxa using transmitted light microscopy, with plate numbers and species codes referenced in Supplementary Table 1. The full reference list for species identifications for both diatoms and soft-bodied algae appears in Supplementary Data Sheet 3.

**FIGURE 4:**
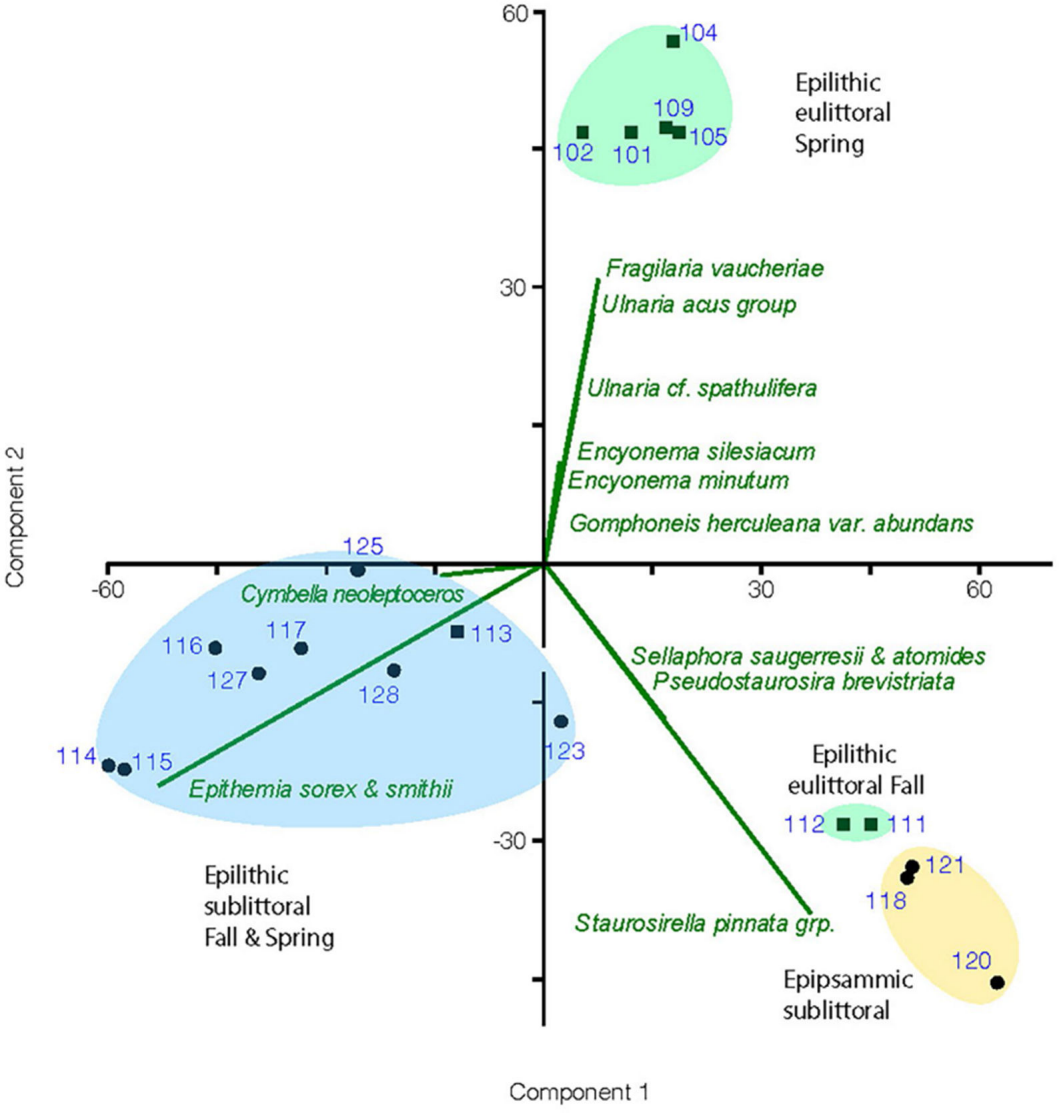
Biplot of principal component analysis for enumerated diatom samples showing groupings by habitat and season of samples and a vector overlay of the diatom species most responsible for the variation in assemblage composition between groupings. Sample numbers are the UNRMNH catalog # found in Table 1. Eulittoral samples are marked with a square symbol and sublittoral samples with a circle.

**FIGURE 5:**
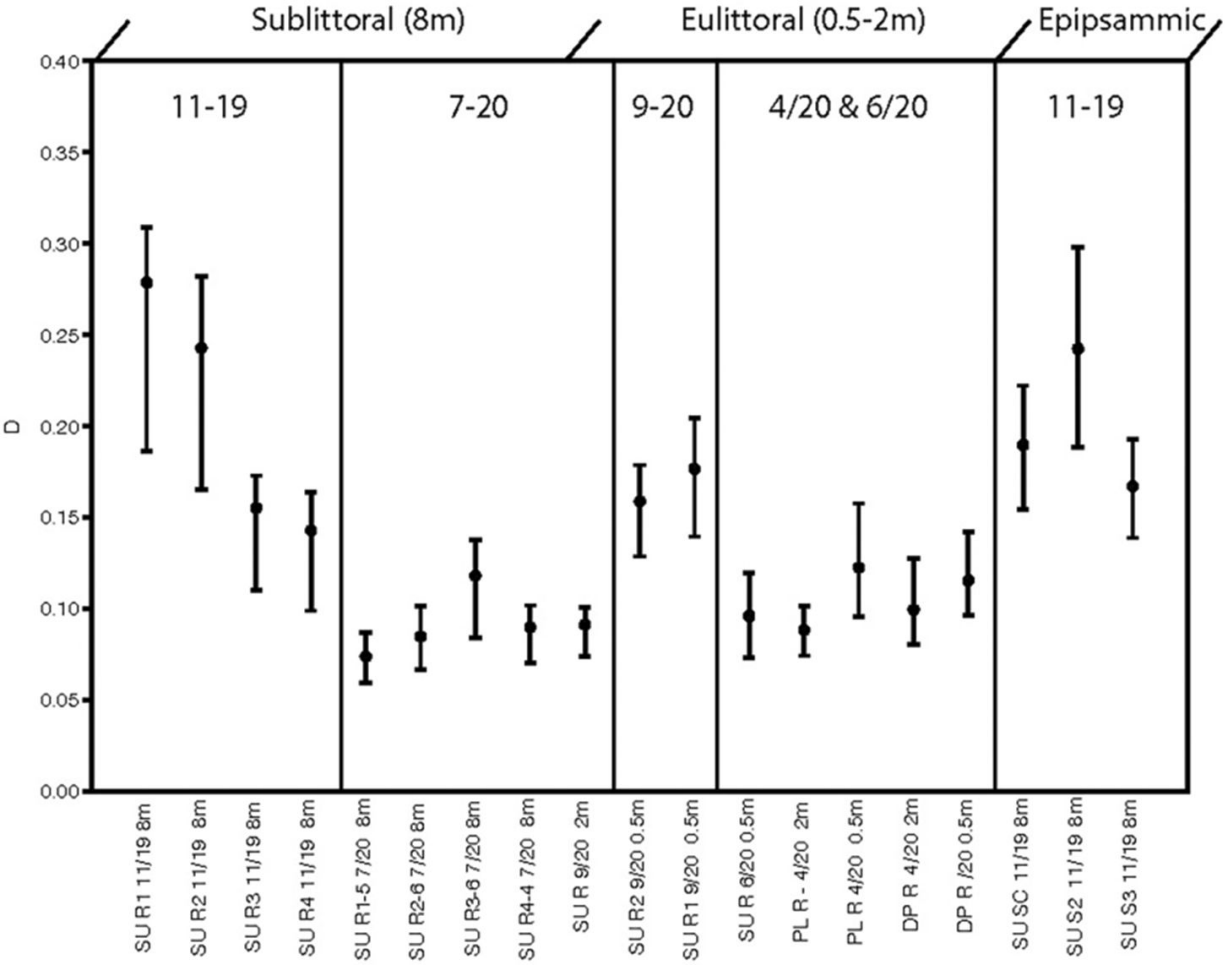
Plot of species dominance, D in enumerated diatom samples.

Results of the PCA performed on the cleaned diatom sample data are illustrated in a biplot (Figure 4). The first 2 axes of this PCA were found to be significant accounting for 43 and 31% of the variance, respectively. Diatom species composition showed distinct groupings between epilithic and epipsammic communities, as well as between the Spring eulittoral and the sublittoral assemblages. The species which accounted for the largest differences are plotted as a vector overlay. Alpha diversity indices were also calculated in PAST, showing a range in species richness, S, from 22 to 36, and species dominance, D, from 0.07 to 2.7 (Figure 5). These values can be found in Supplementary Table 2. In PAST, D is calculated as 1-Simpson (Hammer et al., 2001), with higher numbers indicating a more uneven sample dominated by a particular species. These communities are discussed in further detail below.

#### 3.1.1. Eulittoral zone flora

For the epilithic samples, there was a sharp distinction in the composition of the eulittoral samples (0.5–2 m depth) versus the sublittoral samples, as described in previous investigations. The eulittoral zone contained a mix of diatoms and soft-bodied algae. Diatoms represented between 42 and 93% of the enumerated cells from Spring samples, and 56–66% of the September samples, with the remainder of the flora composed of filamentous and coccoidal cyanobacteria, and green algae. Common taxa observed were *Calothrix, Leptolyngbya, Phormidium, Schizothrix*, *Gloeothece* and *Chroococcus* (Table 3). Common diatoms that typified the eulittoral epilithic habitats were species of fragilarioids, gomphonemoids, cymbelloids and epithemioids, including *Gomphoneis herculeana* var. *abundans* Kociolek and Stoermer, *Gomphoneis eriense* var. *rostrata* Kociolek and Stoermer, *Gomphoneis eriense* var. *angularis* Kociolek and Stoermer, *Encyonema minutum* (Hilse) D.G. Mann and *E. siliacum* (Beisch) D.G. Mann, *Cymbella neocistula* group, *Ulnaria cf. ulna var. spathulifera* (Grunow) Aboal, *Fragilaria vaucheriae* (Kützing) Peterson, *Synedra mazamaensis* Sovereign, *Ulnaria acus* group, and FRAG01-02 species complex. These taxa were particularly prominent in the Spring samples. In the September samples, chains of *Pseudostaurosira* and *Staurosirella* became more prominent. *Epithemioids* were near absent in the Spring samples, but became prominent in the eulittoral zone in the Fall.

#### 3.1.2. Sublittoral zone flora

Epilithic samples from the November 2019 and July 2020 samplings at Sunnyside that were taken in the sublittoral zone, were characterized in previous studies as a flora dominated by nitrogen-fixing cyanobacteria (*Calothryx* and *Tolypothrix*), with diatoms as the second most important group (Loeb and Reuter, 1984; Atkins et al., 2021). Our data show that diatoms represented the bulk of the biovolume relative to the soft-bodied algae, ranging from 58 to 100% of the biovolume. This high abundance persisted throughout the transects from 8 m to 50 m, regardless of substrate or season. Using valve counts, diatoms represent a smaller percentage, but were still the dominant algal group in most samples, ranging from 30 to 93%. The discrepancy between the valve and biovolume percentages is attributed to the difference in cell size where the diatom cells have an overall larger biovolume. The soft-bodied community was predominantly cyanobacteria, including the filamentous and coccoidal forms observed in the eulittoral zone (Table 3). No seasonal difference was observed in the composition of the soft-bodied taxa. However, differences by substrate and depth were observed in that the filamentous branching cyanobacterial filaments that were epilithic were found in lesser abundance with depth. The diatom fraction was dominated by species of *Epithemia* (largely *Epithemia sorex* Kützing, but also *E. adnata* (Kützing) Brébisson, *E. gibba* (Ehrenberg) Kützing, *and E. turgida* var. *westermannii* (Ehrenberg) Grunow), ranging from 18 to 44% of the valves counted in a given sample. *Epithemia* spp. were more abundant in the November, 2019 samples than the July, 2020 samples (mean abundance of 27% valves counted in November 2019, and 20% in July, 2020). Another abundant species amongst the epilithic samples was *Cymbella neoleptoceros*, ranging from 2 to 13% of the valves counted in a given epilithic sample.

#### 3.1.3. Epipsammic habitat

The epipsammic samples taken from surface sediment represent a different habitat for diatoms and have a markedly different proportion of taxa in the assemblage, despite its proximity to the rock samples in the same plots. In addition, sediment samples may represent a mixed assemblage of diatoms living *in-situ* in the sediment surface, plus washed in diatoms from nearby habitats. The % dead diatoms was higher in epipsammic samples (ranging from 42 to 62%) than epilithic samples (ranging from 11 to 37%). The epipsammic samples counted were dominated by colonial fragilarioid chains, mostly belonging to *Staurosirella pinnata* group. *S. confusa* Morales, *S. martyi* (Héribaud) E. Morales and Manoylov, and *Pseudostaurosira brevistriata* (Grunow) D.M. Williams and Round, and FRAG01, a taxon morphologically close to *Fragilariforma virescens* (Ralfs) Williams and Round. Interestingly, *Epithemia* and *Encyonema* were rare in these samples, despite the proximity to rocks covered in these taxa, and indicates a strong habitat partitioning in the diatoms by substrate. Soft-bodied algae also showed strong habitat distinctions. The attached filamentous branched cyanobacteria, typical in the epilithic samples, like *Calothrix*, were generally absent in the epipsammic samples.

## 4. Discussion

### 4.1. Comparison with 1960’s–80’s surveys

In the early surveys, Goldman (1974) identified 60 species of diatoms in his periphyton samples. Of the 115 taxa observed in the Voucher flora, 23 can be harmonized with taxa in Goldman’s list, and another 3 tentatively harmonized. Of the remaining taxa another 27 have sufficiently enduring and stable species concepts to confidently conclude that they were not reported by Goldman during his surveys. The remaining taxa, roughly 1/3 of those we observed, could not be reconciled with Goldman’s species lists, and may in fact have been observed in the 1970’s. Our survey, while encompassing fewer years and fewer samples, was able to recognize roughly 2-fold the number of species than was observed by Goldman (1974). This does not necessarily indicate that biodiversity today is that much greater than it was in the early 1970’s, but may rather reflect methodological differences. In our study, we used permanent mounts of cleaned siliceous material, whereas Goldman (1974) used inverted light microscopy on settled live material. Our method allows for more extensive scanning of strewn slides at higher magnification, that may allow for the identification of more species than with live material using the Utermöhl method used by Goldman’s group. Many of the species we documented were rare and did not show up in the enumerations, save for 1 or 2 valves.

As is typical of diatom communities, there is a strong seasonal component as well as high species dominance during times of rapid growth, such as during Spring bloom. This was observed in Goldman’s 1971 growth experiments on glass cylinders set up in the sublittoral zone. Goldman (1974) found that in both the Summer and Winter, the majority of the taxa were represented by ~8 species each season, but the species composition differed. Summer samples showed roughly equal dominance between *Mougeotia* green algae and diatoms *Epithemia argus* (Ehrenberg) Kützing, *E. gibba*, *Synedra ulna* (Nitsch) Ehrenberg, *Cymbella ventricosa* Agardh, *Navicula aurora* Sovereign, and *Fragilaria capucina* Desmazières. In contrast, the Winter samples were dominated by *Fragilaria actinastroides* Lemmermann, *Fragilaria crotonensis* Kitton, *Gomphonema parvulum* Kützing, *Lindavia bodanica* (Eulenstein ex Grunow) Nakov, Guillory, Julius, Theriot and Alverson, *Synedra ulna*, *Gomphoneis herculeana*, and *Melosira crenulata* [possibly = *Aulacoseira pusilla* (Meister) Tuji and Houki]. These observations differ substantially from our observations of the most dominant species. Notably less green algae was present, and there was a greater abundance of the *Epithemia*-cyanobacteria assemblage components, especially in November samples. Community analysis from studies in the 1980’s provides less species-level data, but still indicates there was a significant green algae component during that time. The eulittoral habitat in the 1980’s was dominated by *Gomphoneis*-*Synedra* diatoms and green algae, whereas the sublittoral zone (>2 m) was dominated by nitrogen fixing cyanobacteria (Loeb and Reuter, 1984; Aloi et al., 1988; Atkins et al., 2021). By comparison, our data show cyanobacteria to be more prominent in both eulittoral and sublittoral zones, and green algae much less abundant. The 1980’s community observations showing distinctions between the eulittoral and sublittoral zones still persist today with stalked diatoms abundant in the eulittoral zone. Dominant seasonal species observed in the Goldman 1970–71 experiments differ from the 2019–20 data, yet the explanation for these differences cannot be attributed to a specific factor. The data are merely 2 annual snapshots spaced 50 years apart, and may in part reflect interannual variation, seasonal differences, and differences in natural versus artificial substrate. None-the-less, the differences are noted. Of the dominant species observed in the sublittoral zone by Goldman (1974), nearly all have been surpassed by other species.

### 4.2. Community analysis

#### 4.2.1. Assemblage composition and seasonality

##### 4.2.1.1. Soft algal community

Algal community ecology information, including potential toxicity, and genus/species function in habitats, can be inferred from the observed morphological traits (filamentous, heterocystous, motile, stalked, monoraphid, biraphid) and growth forms (colonial, unicellular, planktonic, benthic; Stevenson et al., 2010). The abundance of heterocytes of cyanobacteria are indicators of nitrogen fixation (Porter et al., 2008). Algal groups summarized in proportions could be used as a food web indicator, because invertebrate grazers have strong preferences for diatoms (unicellular and stalked growth forms) versus other algal groups (Wellnitz and Poff, 2012; Stevenson, 2014). Cell and biovolume counts have been found to be complementary in the information they produce. Biovolume is a good tool for understanding community structure and biomass changes. Meanwhile, cell relative abundance was found to be better than biovolume in its correlation environmental factors, such as nutrient concentrations (Lavoie et al., 2006).

##### 4.2.1.2. Fragilarioid-gomphonemoid assemblage

Eulittoral samples were largely inhabited by an assemblage of stalked gomphonemoid diatoms and large elongate solitary fragilarioids that showed a strong seasonal variation. The highest numbers of gomphonemoids, *Ulnaria* spp. and *Fragilaria vaucheriae* occurred in the Spring, decreasing in the Fall. Spring samples were also favored by the smaller *Encyonema* species, *E. minutum* and *E. silesiacum*. Fall samples showed increased numbers of small biraphids such as *Adlafia* and *Sellaphora*, monoraphids, and colonial fragilarioid chains (Table 3). As a result, the Sunnyside Fall eulittoral samples inhabited by this flora plot separately from the Spring eulittoral samples on the PCA biplot, and were grouped closer to the epipsammic samples (Figure 4). One possible explanation for this result is that the colonial fragilarioid chains form chains or ribbons that passively tangle amongst periphyton, but can also be entrained in the water column and move between epilithic and epipsammic habitats. Likewise, small biraphid species are solitary motile species that prefer moving water, and can move between the epipsammic and epilithic communities (Table 4).

##### 4.2.1.3. Epithemioid-dominated assemblage

The epithemioid-dominated assemblage occurs largely in the sublittoral zone epilithic habitats. At Sunnyside, it occurred only in the 0.5 m samples, as is illustrated through enumeration of a 2 m September 2020 sample, which plotted with the sublittoral samples in the PCA biplot (Figure 4). Seasonal variance in the epithemioid-dominated community was less apparent than with the *Ulnaria*-gomphonemoid dominated community in the eulittoral zone. Spring and Fall sublittoral rock scrape samples grouped together in the PCA biplot, represented by greater abundances of *E. sorex*, *E. smithii* Carruthers and *Cymbella neoleptoceros* (Figure 4). Smaller variations in Spring and Fall composition can be seen in the abundance count summaries (Table 4). The strong seasonal preference in gomphonemoids seen in the eulittoral zone was also manifested in sublittoral counts to a lesser extent. They were present in minor amounts in the sublittoral zone (1–6% of valve counts) in the July 2020 enumerations (i.e., *Gomphonema pratense* Lange-Bertalot and Reichardt, *Gomphosinica geitleri* (Kociolek and Stoermer) Kociolek, You, Wang, and Liu, *Gomphoneis eriense* var. *rostrata*), but were near absent (<1%) in the Fall 2019 samples.

All the *Epithemia* species are benthic, low nitrogen (N) specialists, and indicative of N-limited environments, as they host endosymbiotic N-fixing spheroid bodies derived from cyanobacteria (DeYoe et al., 1992; Nakayama et al., 2011). These species are also considered to be highly sensitive to human disturbance and ecologic degradation, associated with good water quality (score of 2 on Biological Condition Gradient in Paul et al., 2020, Table 4). *Epithemia* species are all moderately motile, solitary forms attaching prostrate to the substrate *via* mucilage. The high abundance of *Epithemia* supports the soft-bodied algal data, showing that cyanobacteria was a large component of the algal population. *Cymbella neoleptoceros* is also a slightly motile solitary benthic form. In boil mounts it was found in close association with *Epithemia* and filamentous cyanobacteria. *Encyonema* species are slightly motile, forming mucilage sheaths or tubes and may be solitary or extend in long chains (Spaulding et al., 2021). Tube forming diatoms like *Encyonema* and *C. neoleptoceros* are in a high profile guild with taller stature (Rimet and Bouchez, 2012) and can add to the thickness of the biomass attached to the rock surface.

#### 4.2.2. Species diversity

Alpha diversity indices calculated from the cleaned diatom samples and run in PAST show variation in species richness (i.e., number of taxa/sample), ranging from 22 to 36 taxa. This number is not terribly informative nor accurate because most of the species are rare, with a low chance of appearing in the enumerations. Scanning of these slides showed much larger numbers of the entire 115 OTUs are in fact present in a given sample, and the many rare taxa were overshadowed by a few dominant species. Different methods are necessary to approach true species richness through enumeration (Tyree et al., 2020b), and were beyond the scope of this present work. A more informative metric is species dominance, D, which ranges from 0 to 1. A D = 0 indicates all taxa are equally present, whereas 1 indicates a species dominates the sample completely. For the sublittoral zone, there was higher species dominance in the Fall epilithic samples at 0.5 m compared to Spring (Figure 5). At Sunnyside, the *Epithemia*-cyanobacteria dominated assemblage shoaled into the base of the eulittoral zone, as is shown in the PCA biplot mentioned in the previous section (Figure 4), as well as with the diversity index. At Sunnyside, the September 2020 2 m Sunnyside sample has lower D value then the 0.5 m samples, similar to the 8 m sublittoral samples from this location (Figure 5). While Fall and Spring samples plotted similarly in the PCA multivariate space (Figure 4) based on composition, they separated based on dominance of epithemioids, especially *E. sorex* and *E. smithii* (Table 4). There was also a high dominance in the Fall epipsammic samples, reflective of high numbers of small *Sellaphora* and fragilarioid chains. Diatoms are known to have strongest growth during the Spring. It was therefore anticipated that species dominance would be greater during the July sampling versus the November sampling. However, this was not the case; there was a lower species dominance in July that may reflect the high level of adaption of the sublittoral epithemioid-cyanobacteria assemblage of low nutrient N-fixing specialists as the season progressed. The Fall eulittoral and epipsammic communities showed higher dominance (Figure 5), but dominant species were *Sellaphora* and colonial fragilarioid chains. No explanation can presently be given for this increased dominance, and it will require further investigation.

#### 4.2.3. Implications for management

The degradation of nearshoe habitats in relatively pristine lake waters has been of increasing concern in the last decade (Vadeboncoeur et al., 2021) with increased obsevations from many of the world’s iconic lakes. A major challenge in understanding nearshore water quality degradation resulting from benthic biofilms and metaphytic growth is that rarely do we have an understanding of the taxonomy of the community, and often no understanding of the growth phases of dominant taxa. If we are going to understand and manage nearshore eutrophication in the 21^st^ century, then we need to promote and enhance taxonomic level identifications in lake monitoring programs and rapid response studies during algal blooms. Another challenge in long-term monitoring programs of critical water bodies is maintaining taxonomic consistency amongst the datasets (e.g., Kovalenko and Reavie, 2022; Riato et al., 2022). In the Lake Tahoe basin, as more agencies and research groups become involved, these challenges will grow and are met best through the analytical methods that result in taxonomically consistent practices. Our use of a diatom voucher flora for the most speciose part of the algal assemblages provides a means of consistency between projects and research groups, providing it is adopted. Adopting a voucher flora approach earlier in assessment programs can prevent the need for resource intensive post-hoc harmonization, which may require re-analysis of slides and loss of species information when taxa cannot be reconciled (Potapova et al., 2022). Our voucher flora shows a 2-fold level of species diversity than was previously documented, most likely because of the methods involved in examining the diatom fraction through cleaned permanent mounts. As additional samples are analyzed from other parts of the nearshore, we expect the biodiversity to grow. As such the voucher flora can become a living document which is expanded and updated periodically and provides an opportunity for open-source based collaborations among scientists to discuss the importance of taxa in driving changes to ecosystem level properties.

## 5. Conclusion

Herein algal survey and enumeration of diatoms, the most speciose group in the Lake Tahoe nearshore assemblages, is documented and enumerated by the construction of a voucher flora. These data were then combined with taxonomy and enumeration of algal samples preserved in Lugol’s solution collected from 0.5 to 50 m depth, to provide data on the whole algal community. This method captures detailed snapshots of the biodiversity and community structure of nearshore algae epilithic and epipsammic habitats. Additionally, this method provides a means of reconciling taxonomic assignments in any future diatom-based work in the basin, a necessary function in long-term monitoring of ecosystems.

Despite challenges in harmonizing these data with past species lists, it is clear that several prominent taxa have continued to dominate the nearshore since cultural eutrophication and algal blooms became problematic. These include a fragilarioid-gomphonemoid dominated assemblage in the eulittoral zone, and an epithemioid dominated assemblage in the sublittoral zone. Seasonal changes were noted, and the epithemioid assemblage at Sunnyside Harbor impinged into the eulittoral zone seasonally in the Fall with the expansion of cyanobacterial blooms. Significant differences were also seen between epilithic and epipsammic sublittoral assemblages, despite their being spatially adjacent, and underscores the importance of substrate in establishing the algal communities. Comparison between the 1970–71 and 2019–20 taxonomic data, while representing only limited snapshots that are 50 years apart, show substantial changes in the nearshore sublittoral community. Such changes include a decrease in the green alga *Mougeotia*, increases in *Epithemia*, and shoaling of the *Epithemia*-cyanobacteria assemblage to 2 m at Sunnyside. Of the 13 most common taxa in 1970–71, only 1 remains relatively common in the sublittoral zone, *Epithemia gibba*. These data will play a role in continued assessment of algal biodiversity and the overall health in the nearshore environment, providing a means of gauging future biodiversity change from the present conditions.

## Supplementary Material

Supplement1

## Figures and Tables

**TABLE 1:** List of samples taken for diatom analysis.

Catalog #	Sample ID with date	Location	Sample type	Zone	Enumerated
UNRMNH-101	PL-R 4/19 0.5 m	Pineland	rock scrape	eulittoral	y
UNRMNH-102	PL-R 4/19 2 m	Pineland	rock scrape	eulittoral	y
UNRMNH-103	PL-R 4/19 8 m	Pineland	rock scrape	sublittoral	n
UNRMNH-104	DP-R 4/19 0.5 m	Dollar Point	rock scrape	eulittoral	y
UNRMNH-105	DP-R 4/19 2 m	Dollar Point	rock scrape	eulittoral	y
UNRMNH-106	DP-R 4/19 8 m	Dollar Point	rock scrape	sublittoral	n
UNRMNH-107	PL-S 7/19 1.5 m	Pineland	Ponar surface sample	eulittoral	n
UNRMNH-108	PL-S 7/19 8-8 m	Pineland	Ponar surface sample	sublittoral	n
UNRMNH-109	SU-R 6/20 0.5 m	Sunnyside	rock scrape	eulittoral	y
UNRMNH-110	SU-R 6/20 2 m	Sunnyside	rock scrape	eulittoral	n
UNRMNH-111	SU-R1 9/20 0.5 m	Sunnyside	rock scrape	eulittoral	y
UNRMNH-112	SU-R2 9/20 0.5 m	Sunnyside	rock scrape	eulittoral	y
UNRMNH-113	SU-R 9/20 2 m	Sunnyside	rock scrape	eulittoral	y
UNRMNH-114	SU-R1 11/19 8 m	Sunnyside	rock scrape	sublittoral	y
UNRMNH-115	SU-R2 11/19 8 m	Sunnyside	rock scrape	sublittoral	y
UNRMNH-116	SU-R3 11/19 8 m	Sunnyside	rock scrape	sublittoral	y
UNR-MNH117	SU-R4 11/19 8 m	Sunnyside	rock scrape	sublittoral	y
UNR-MNH118	SU-SC 11/19 8 m	Sunnyside	rock scrape	sublittoral	y
UNR-MNH119	SU-S1 11/19 8 m	Sunnyside	surface sed scrape	sublittoral	n
UNRMNH-120	SU-S2 11/19 8 m	Sunnyside	surface sed scrape	sublittoral	y
UNRMNH-121	SU-S3 11/19 8 m	Sunnyside	surface sed scrape	sublittoral	y
UNRMNH-122	SU-S4 11/19 8 m	Sunnyside	surface sed scrape	sublittoral	n
UNRMNH-123	SU-R1-5 7/20 8 m	Sunnyside	rock scrape	sublittoral	y
UNRMNH-124	SU-R1-6 7/20 8 m	Sunnyside	rock scrape	sublittoral	n
UNRMNH-125	SU-R2-6 7/20 8 m	Sunnyside	rock scrape	sublittoral	y
UNRMNH-126	SU-R2-7 7/20 8 m	Sunnyside	rock scrape	sublittoral	n
UNRMNH-127	SU-R3-6 7/20 8 m	Sunnyside	rock scrape	sublittoral	y
UNRMNH-128	SU-R4-4 7/20 8 m	Sunnyside	rock scrape	sublittoral	y
UNRMNH-129	SU-R4-5 7/20 8 m	Sunnyside	rock scrape	sublittoral	n

All samples were processed and permanent strewn slides made. All were examined for taxonomic purposes to construct the voucher flora, and a subset of slides were then enumerated for community analysis. Sample ID with date corresponds is in the format: site location-sample type (R = rock vs. S = sediment)-month/year sampled-depth. See Figure 1 for site locations.

**TABLE 2:** List of samples examined for soft-bodied algal taxonomy and enumerated for algal biovolume (soft-bodied and diatoms combined).

sample ID	Depth	Date coll	Sample type	Zone	Enumerated
SU R1	0.5 m	10-03-2020	rock scrape	eulittoral	y
SU R1	2 m	09-03-2020	rock scrape	eulittoral	y
SU R1	8 m	10-03-2020	rock scrape	sublittoral	y
SU R2	50 m	09-03-2020	rock scrape	sublittoral	y
SU R1	0.5 m	01-06-2020	rock scrape	eulittoral	y
SU R2	8 m	01-06-2020	rock scrape	sublittoral	y
SU R2	20 m	01-06-2020	rock scrape	sublittoral	y
SU R1	0.5 m	29-09-2020	rock scrape	eulittoral	n
SU R1	2 m	29-09-2020	rock scrape	eulittoral	y
SU Sed3	2 m	10-03-2020	rock scrape	eulittoral	y
SU Sed1	30 m	10-03-2020	rock scrape	sublittoral	y
SU Sed2	2 m	29-09-2020	rock scrape	eulittoral	y
SU Sed1	50 m	29-09-2020	rock scrape	sublittoral	y

**TABLE 3:** Summary counts of algae and their habitats from Lugol’s samples.

		sample date:	10-03- 2020	09-03- 2020	10-03- 2020	10-03- 2020	10-03- 2020	09-03- 2020	01-06- 2020	01-06- 2020	01-06- 2020	29-Sep	29-Sep	29-Sep	29-09- 2020
		substrate:	rock	rock	sediment	rock	sediment	rock	rock	rock	rock	rock	rock	sediment	sediment
		Location & depth	SU R 0.5m	SU R 2m	SU S 2m	SU R 8m	SU S 30m	SU R 50m	SU R 0.5m	SU R 8m	SU R 20m	SU R 0.5m	SU R 2m	SU Sed 2m	SU S 50m
Lake Tahoe SOFT algae codes	Type	Dead diatoms	34	88	306	84	475	160	109	165	91	16	86	216	380
		Live diatoms	289	252	292	213	291	272	286	280	281	183	257	297	291
BULBsp1	Green Algae	Bulbochaete	0	11 (14628)	0	24 (31915)	0	0	0	0	0	0	0	0	0
CALepiph	Cyanobactria	*Calothrix epiphytica*	0	0	0	38 (4475)	0	0	0	0	34 (4004)	0	0	0	0
CALfusca	Cyanobactria	*Calothrix fusca*	0	0	0	28 (7700)	0	0	0	0	0	0	0	0	0
CALsp	Cyanobactria	*Calothrix*	0	0	0	0	0	0	0	16 (3140)	24 (4710)	0	0	0	0
CALsp1	Cyanobactria	*Calothrix*	0	31 (6084)	0	0	0	0	0	0	0	0	0	0	0
CALsp4	Cyanobactria	*Calothrix*	0	0	0	37 (&261)	0	0	0	0	0	0	0	0	0
CALsp8	Cyanobactria	*Calothrix*	0	28 (5459)	0	0	0	0	0	0	0	0	0	0	0
CHLORcol	Green Algae	Green coccoid colony	0	0	0	38 (2486)	0	0	0	0	0	0	0	0	0
CHRminim	Cyanobactria	*Chroococcus minimus*	0	0	0	0	0	0	0	0	0	0	64 (2144)	0	0
CHRsp	Cyanobactria	*Chroococcus* sp.	0	32 (1526)	0	0	0	0	0	56 (2671)	0	0	0	0	0
CYAFiH6	Cyanobactria	Unknown Cyanophyte filament (heterocytes) # 6	0	0	0	0	0	0	0	0	0	0	0	12 (3391)	0
CYAFiH7	Cyanobactria	Unknown Cyanophyte filament (heterocytes) sp#7	0	0	0	0	0	0	0	0	0	0	0	0	2
CYAFILH	Cyanobactria	Unknown Cyanophyte filament (heterocytes)	0	0	0	142 (6967)	0	56 (2748)	0	24 (1178)	86 (4219)	0	48 (2355)	0	0
DESabun	Green Algae	*Desmodesmus cf abundans*	0	0	12 (3041)	0	0	0	0	0	0	0	0	0	0
DICHsp1	Cyanobactria	*Dichothrix* sp.	0	0	0	18 (3533)	0	0	0	0	0	0	0	0	0
GLOdubia	Green Algae	*Gloeothece dubia*	0	0	0	0	0	0	0	2	0	0	0	0	0
*GLOmemb1*	Green Algae	*Gloeothece membranacea var 1*	0	0	0	21 (2375)	0	0	0	0	0	0	0	0	0
*GLOmembr*	Green Algae	*Gloeothece membranacea*	0	0	0	125 (33510)	0	0	0	0	0	0	0	0	0
LEIspp	Cyanobactria	*Leptolyngbya* sp.	0	24 (3014)	0	0	0	0	0	0	0	0	31 (274)	0	0
LEPsp1	Cyanobactria	*Leptolyngbya* sp. 1 ?	75 (662)	68 (601)	11 (97)	27 (238)	12 (106)	24 (211)	0	0	27 (238)	94 (830)	0	0	24 (212)
LEPsp3	Cyanobactria	*Leptolyngbya* sp. 3?	0	0	0	0	0	0	37 (581)	0	0	0	0	0	0
MERsp	Cyanobactria	*Merismopedia* sp.	0	0	0	0	0	0	0	0	0	0	32 (2048)	0	0
MOUsp1	Green Algae	*Mougeotia* sp. 1 ?	0	0	0	0	0	0	0	0	0	0	4 (20347)	0	0
OEDsp	Green Algae	*Oedogonium* sp.	0	12 (22608)	0	0	0	0	0	0	0	0	0	0	0
OSCsp1	Cyanobactria	*Oscillatoria* sp. 1 ?	0	0	0	0	0	0	0	0	0	0	0	3 (1808)	0
PEDborya	Green Algae	*Pediastrum boryanum*	0	0	0	0	12 (6120)	0	0	0	0	0	0	0	12 (6120)
PHOsp	Cyanobactria	*Phormidium* sp.	0	0	10 (981)	0	14 (1374)	33 (3238)	0	0	0	0	0	2	0
PHOsp.1	Cyanobactria	*Phormidium* sp. 1 ?	0	0	0	0	0	0	0	0	0	0	24 (2355)	0	0
PHOsp2	Cyanobactria	*Phormidium* sp. 2	0	14 (1374)	0	0	0	0	0	0	0	0	0	0	0
Plagprol	Cryptophyte	*Plagioselmis prolonga* Butcher	0	0	0	0	0	0	0	0	0	0	0	0	1(180)
PLEtrab	Green Algae	*Pleurotaenium* cf. *trabecula*	0	1	0	0	0	0	0	0	0	0	0	0	0
SCEecorn	Green Algae	*Scenedesmus ecornis*	0	0	2	0	0	0	0	0	0	0	0	0	0
SCHsp	Cyanobactria	*Schizothrix* sp.	0	0	0	0	0	0	0	48 (848)	0	0	0	0	0
Schsp1	Cyanobactria	*Schizothrix sp.1*	0	0	0	0	0	0	0	0	0	0	0	0	0
SCHsp2	Cyanobactria	*Schizothrix* sp. 2	0	0	0	0	0	0	0	0	26 (459)	0	0	0	0
SCHsp3	Cyanobactria	*Schizothrix* sp. 3	0	118	0	0	0	0	0	0	0	0	0	0	0
STAsp2	Green Algae	*Staurastrum* sp. 2 ?	0	0	0	2	0	0	0	0	0	0	0	0	0
TRAsp	Euglenoids	*Trachelomonas* sp.	0	0	0	0	0	0	0	0	0	0	0	0	3 (3391)
UNKNcoc	UNKNcoc	Unknown Phyla	0	0	0	0	0	0	0	0	0	0	0	0	0
		diatom biovolume	86700	75600	87600	1091625	50925	1632000	85800	1960000	1440125	1830000	2570000	89100	898462.5
		soft biovolume	13586	55294	4119	93460	7600	6197	581	7837	13592	830	29523	5199	9903

Count number and biovolume (in parentheses) are reported. A comprehensive list of observed soft-bodied taxa, including those too rare to show up in the counts, appears in the Supplementary material.

**TABLE 4:** Summary counts of diatoms from seasons and habitats reported as % relative abundance.

Species code (OTU)	Species or counting group name	11-19 mean abund epilithic 8m %	11-19 mean abund sediment 8m%	7-20 mean abund epilithic 8m %	4-23,6-1 mean spring eulittoral 0.5-2m %	9-20 mean fall eulittoral 0.5-2m %	ecological notes	Biological Condition Gradient (Paul et al 2020)	Ecological Guilds (Rimet & Bouchez 2012)
EPI02, 04	*Epithemia sorex & smithii*	36	0	19	1	9	moderately motile, prostrate, solitary, hosts endosymbiotic cyanobacteria, low N specialist	2	motile
STRL02, 03, 04	*Staurosirella pinnata & FLL1*	2	36	8	2	17	non motile vertical attachment, forms chains	4	low profile
PS02	*Pseudostaurosira brevistriata*	2	20	5	2	11	non motile, vertical attachment, chains	3	low profile
FRAG01, 02	*unknown fragilarioid*	9	12	5	4	1	varied	NA	varied
ENC04	*Cymbella neoleptoceros*	8	0	13	3	7	slightly motile solitary attached in mucilage tube, epilithic and epiphytic brooks and lakes in alpine/subalpine settings	NA	high profile
FRAG07	*Fragilaria vaucheriae*	0	1	0	17	1	non motile, forms chains, vertical attachment at one end	3	high profile
FRAG03, 04, 05	*Ulnaria acus group*	0	0	0	15	1	epilithic and epiphytic biofilms in rivers and lake shores	NA	attached
SELL01, 02	*Sellaphora saugerresii & atomides*	2	7	2	0	16	slightly motile unattached solitary	2	motile
ACHN01	*Achnanthidium minutissimum*	2	0	3	7	5	slightl motile prostrate attachment short mucilage stalks or pads, solitary or very short chains	3	low profile
STRL01	*Staurosirella martyi*	3	5	4	2	4	non motile solitary epipsammic, zig-zag colonies rivers and lakes	NA	high profile
ULN01	*Ulnaria cf. spathulifera*	0	0	0	10	0	attached epilithic or epiphytic, spring eulittoral species in Tahoe	NA	attached
ENCP01	*Encyonopsis subminuta*	3	0	7	0	4	moderately motile, prostrate attachment, solitary	3	motile
MONO01	*Monorapphid sp. 1* Tahoe	2	0	4	3	2	NA	NA	low profile
ADL01	*Adlafia suchlandti*	2	0	5	0	1	slightly motile solitary, unattached in rivers and lakes, highest abundance in streams	3	low profile
ENC05	*Encyonema silesiacum*	0	0	0	6	0	slightly motile, tube-forming, flowing water	NA	motile
MAST01	*Mastogloia lacustris*	3	0	3	0	0	moderately motile, unattached, solitary in lakes	NA	motile
EPI05	*Epithemia gibba*	4	0	1	0	1	moderately motile, prostrate, solitary, usually epiphytic, hosts endosymbiotic cyanobacteria, low N specialist	2	motile
PLAN02	*Planothidium frequentissimum*	1	5	0	1	2	attached, adnate (attached via mucilage from raphe)	4	low profile
ENC02	*Encyonema minutum*	0	0	0	5	0	slightly motile, tube-dorming, flowing water	NA	motile
EPI01	*Epithemia adnata*	1	0	4	1	1	moderately motile, prostrate, solitary, usually epiphytic, hosts endosymbiotic cyanobacteria, low N specialist	2	motile
COCC02, 03, 04	*Cocconeis sp.*	0	2	1	1	2	non motile to weakly motile, adnate, solitary	NA	low profile
GOMS03	*Gomphoneis geitleri*		0	0	3	0	non motile mucilage stalks	NA	high profile
NITZ04	*Nitzschia innominata*	0	1	1	3	0	highly motile, unattached, prostrate, solitary, probably indicative of clean water	NA	motile
SRL05	*Staurosirella* sp. 5 Tahoe	0	1	2	1	0	nonmotile, unattached, occasional colonies	NA	low profile
PSAMM02	*Psammothidium levanderi*	0	1	1	0	2	slightly motile, prostrate, solitary on sand grains	2	low profile
GOMS02	*Gomphoneis herculeana var. abundans*	0	0	0	2	0	non motile mucilage stalks	2	high profile
NAVO1	*Navicula cryptotenella*	1	1	1	2	0	moderately motile unattached solitary	NA	motile
SYN01	*Synedra mazamaensis*	0	1	0	1	0	non motile, prostrate attachment, lakes	2	low profile	
NITZ09	*Nitzschia hanzstchiana*	0	0	0	2	0	highly motile, unattached, prostrate, solitary	NA	motile	
GOM07	*Gomphonema pratense*	0	0	2	1	0	non motile mucilage stalks	NA	high profile	
COCC01	*Cocconeis placentula*	1	0	0	0	2	non motile to weakly motile, adnate, solitary	4	low profile	
CAV02	*Cavinula jaernefeltii*	1	1	1	0	0	moderately motile, unattached solitary, may be planktonic, oligotrophic to mesotrophic lakes and rivers	2	motile	
NAV03	*Navicula radiosa*	0	0	1	0	1	moderately motile unattached solitary	3	motile	
EPI03	*Epithemia turgida v. westermannii*	1	0	1	0	1	moderately motile, prostrate, solitary, hosts endosymbiotic cyanobacteria, low N specialist	2	motile	
GOM01	*Gomphonema laticollum*	0	0	0	2	0	non motile mucilage stalks	NA	high profile	
GOMS01	*Gomphoneis eriense var. rostrata & angularis*	0	0	0	1	0	mucilage stalks, eulittoral zone in Tahoe	2	high profile	
CYM01, 03, 04	*Cymbella neocistula grp*	0	0	0	1	0	mucilage stalks, eulittoral zone in Tahoe	4	high profile	
NAV02	*Navicula aurora*	0	1	0	0	0	moderately motile unattached solitary	2	motile	

Only species >1% abundance are reported, and ecological information included. The Biological Condition Gradient 2: Highly sensitive to pollution and disturbance, typically on common; 3: intermediate sensitivity, ubiquitous and abundant in relatively undisturbed areas; 4: indiscriminate, found in a range of conditions, increasing in abundance at disturbed sites.

## Data Availability

The original contributions presented in the study are included in the article/Supplementary materials, further inquiries can be directed to the corresponding author.
