# Supplementary material for: Characterization of algal community composition and structure from the nearshore environment, Lake Tahoe (United States)
Source: Front Ecol Evol. Author manuscript; Available in PMC 2024 Jan 20. (PMC10750852; doi:10.3389/fevo.2022.1053499)
Supplement: Supplement1 [file NIHMS1947911-supplement-Supplement1.zip › Supplementary Data sheet 1.pdf]

Supplementary file Common soft-bodied algae

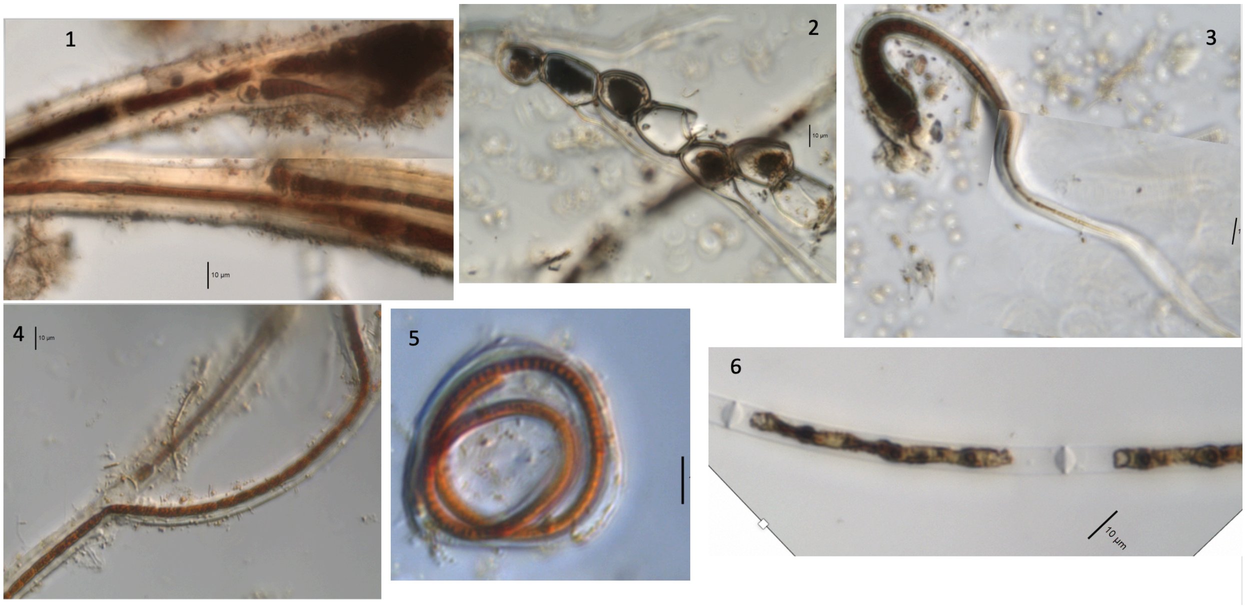

Plate 1. Soft algae images from Lake Tahoe collection 2020, filamentous. 1. *Dichothrix cf. hosfordii,* cells shorter than wide at the base, not to slightly constricted at the cross wall, becoming cylindrical above to long cylindrical in the apical zone. Trichomes 10-16 µm wide at the base, ending in long hair, heterocyst a hemispherical, 7-9 µm in diameter. Sheaths thick, lamellate, several trichomes within a sheath at the base, the branches emerging in their own sheath.; 2. *Bulbochaete sp.1* Lake Tahoe, branching filament, base of non-photosynthetic setae visible; 3. *Calothrix fusca* (Kütz.) Bornet et Flah**.** unbranched single trichome, bulbous at the base, bent, tapering to a distinct hair at the end, cells near the base 12 µm wide, cell lengths 3-4.5 µm, sheath colorless lamellated, heterocyst basal hemispherical diameter 9 µm; 4. *Unknown Cyanophyte filament sp.4* Lake Tahoe*,* filament with heterocytes, false branching, irregular cell outlines, sheath visible; 5. *Limnolyngbya cf. circumcreta* (Anagnostidis & Koma´rek) X. Li & R. Li, trichomes solitary, screw-like coiled, cells more or less quadratic slightly constricted, 1.9-2.3 um long, 1.6–2.4 um wide; visible loose sheath; 6. *Mougeotia sp.2* Lake Tahoe. All scale bars equal 10 µm.

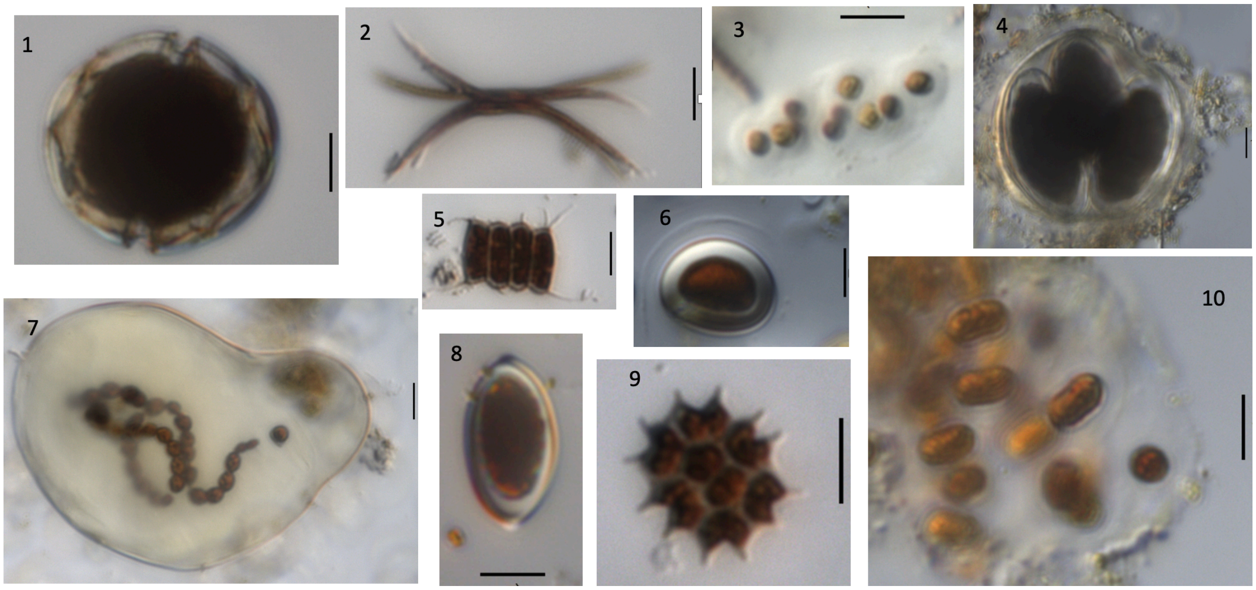

Plate 2. Soft algae images from Lake Tahoe collection 2020 coccoid and colonial. 1. *Parvodinium inconspicuum* (Lemmermann) Carty; 2. *Ankistrodesmus falcatus* (Corda) Ralfs; 3. *Gloeocapsa cf. punctata* Nägeli, connected irregular colonies composed of small, several–celled subcolonies, cells solitary or in small groups, surrounded by up to 5 µm wide gelatinous layer. Cells spherical or slightly elongate 3–45 µm in diameter; 4. *Oocystis sp.1* Lake Tahoe, chloroplast structure hard to see; 5. *Desmodesmus abundans* (Kirchner) E.H. Hegewald, cells arranging linearly, outer cells with long spines at both ends and short spines at outer side; inner cells have a short spine on one or both ends. Cell size 18x5µm; 6. *Chroococcus sp.1* Lake Tahoe, lamellated sheath around each cell, length 13 µm, 3-5 µm in diameter; 7. *Nostoc sp.2* Lake Tahoe, cells 3-5 µm in diameter, developing colony wide well defined mucilage; 8. *Oocystis solitaria* Wittr, cell solitary ovoid, apices broadly rounded with a nodular wall thickening, 13 µm wide, 27 µm long, chloroplasts numerous, with a pyrenoid; 9. *Pediastrum boryanum* (Turpin) Meneghini; 10. *Aphanothece stagnina* (Spreng.) A. Braun, gelatinous irregular microscopic colony, cells homogeneous in content, oval, cylindrical with rounded ends, 7-9 µm long, 4-6 µm in diameter, loosely arranged in colony, each cell with own indistinct envelope. All scale bars equal 10 µm.

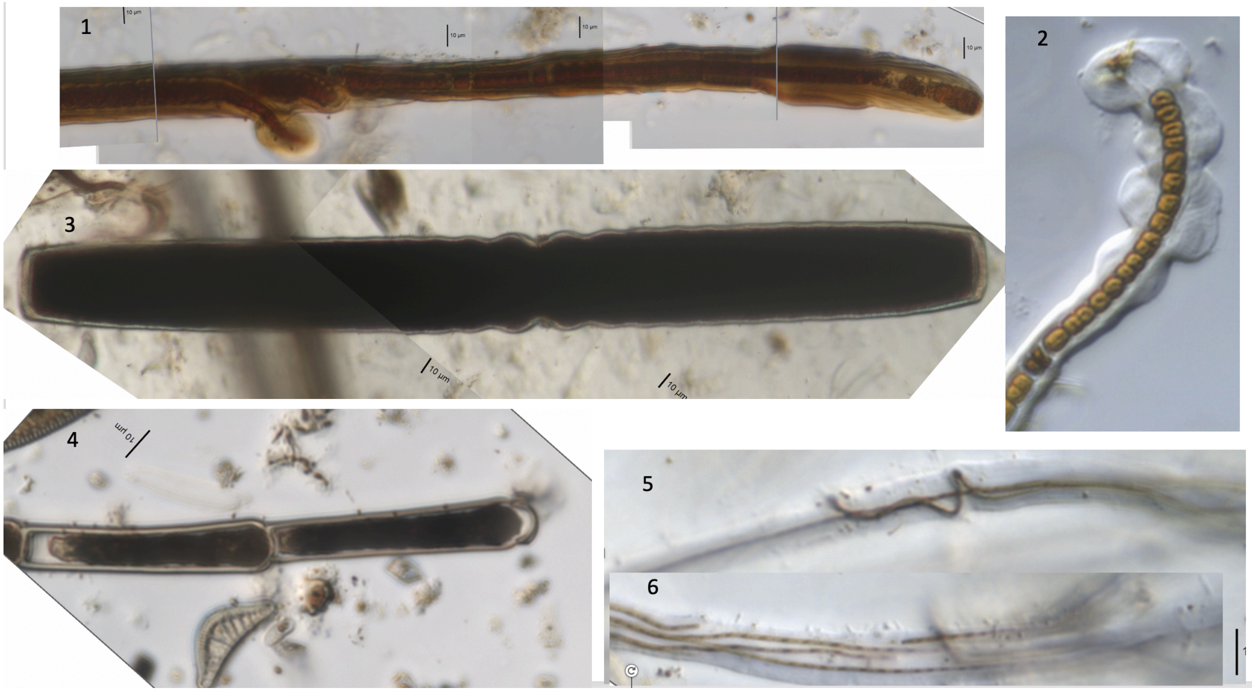

Plate 3. Soft algae images from Lake Tahoe collection 2020 coccoid and filamentous. 1. *Petalonema alatum* (Borzì ex Bornet & Flahault) Correns, false branching lateral, sheaths firm, very thick, distinctly funnel-like lamellated; 2. *Unknown Cyanophyte filament* sp.*2* Lake Tahoe, sheath variable in outline, cell irregular and constricted, no branching or confirmed heterocytes; 3. *Pleurotaenium cf. trabecula* Nägeli diameter 36-42 µm, ends narrower length 340 µm, chloroplast structure not verified; 4. *Oedogonium* sp.1 Lake Tahoe; 5-6. *Schizothrix* sp.3 Lake Tahoe*,* sheaths fine, gelatinous, homogeneous*,* 2 to 4 parallel trichomes within a sheath, longer trichomes straight, flex, or spiral, cell diameter 0.9 to 1.3 µm, cells not constricted*.* All scale bars equal 10 µm, scale bar shown on fig. 5 applicable for figures 2 and 6.
