# Supplementary material for: Characterization of algal community composition and structure from the nearshore environment, Lake Tahoe (United States)
Source: Front Ecol Evol. Author manuscript; Available in PMC 2024 Jan 20. (PMC10750852; doi:10.3389/fevo.2022.1053499)

Plate 1 Centrics

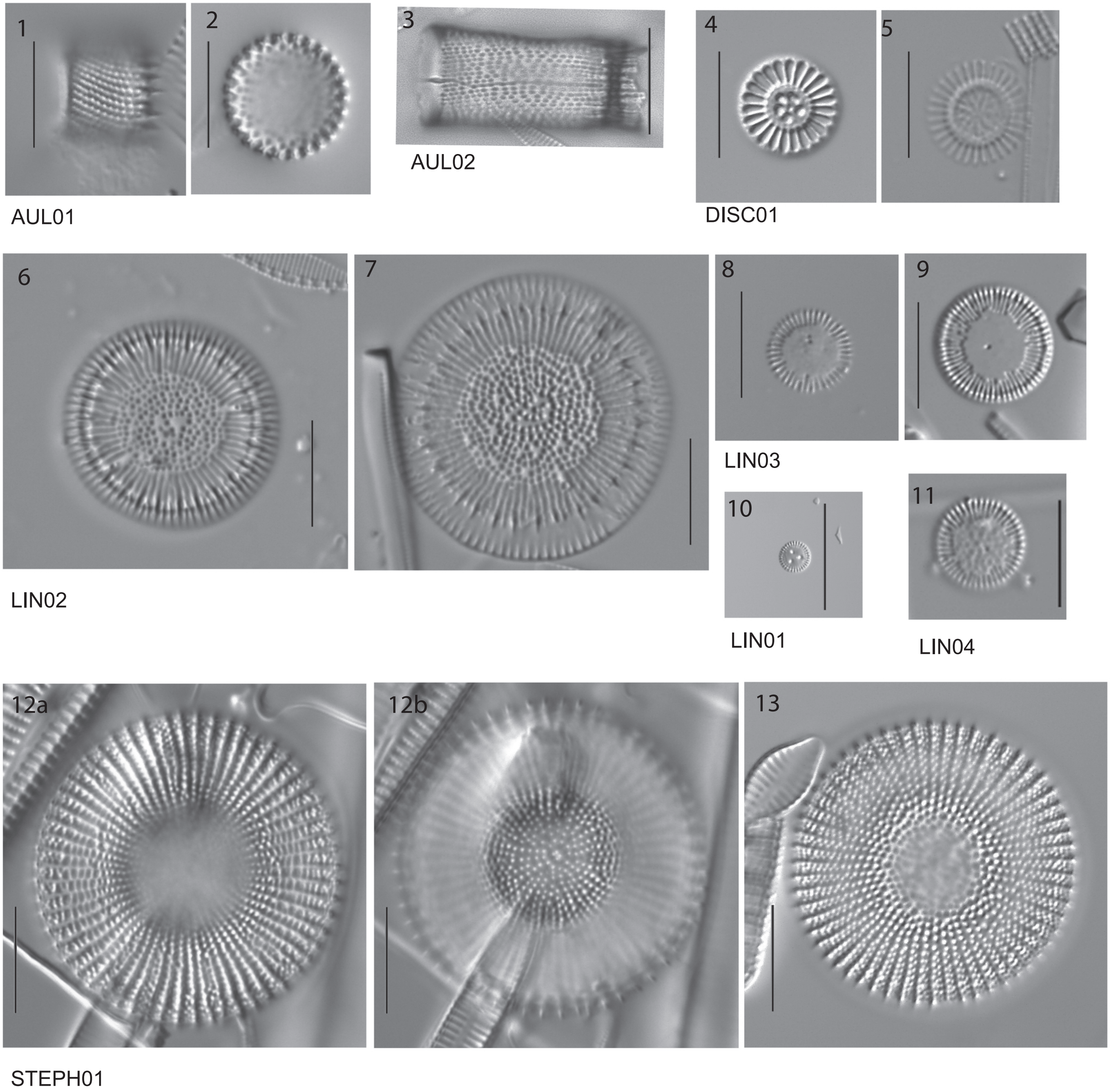

Plate 2 Araphids

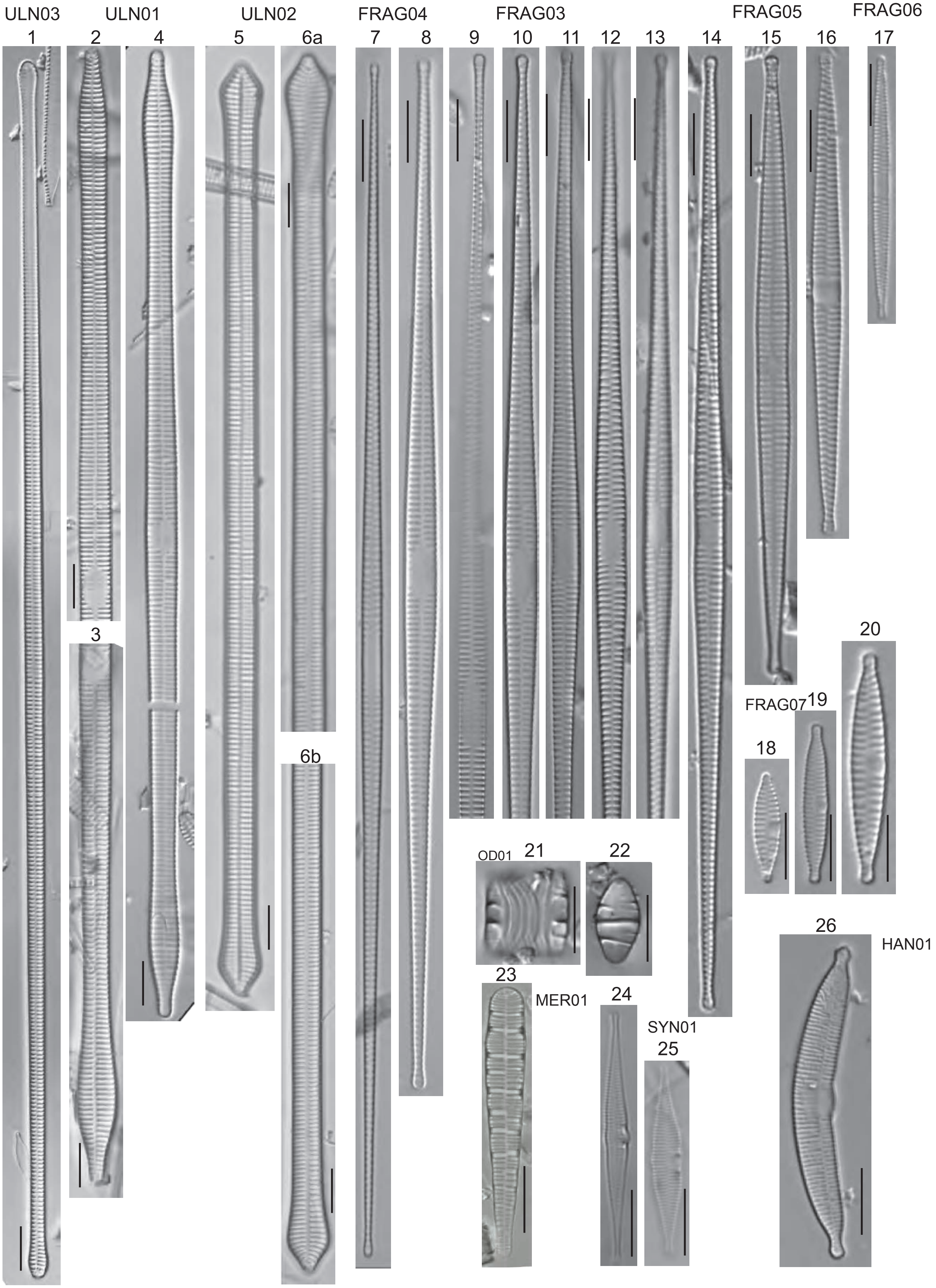

Plate 3 Araphids

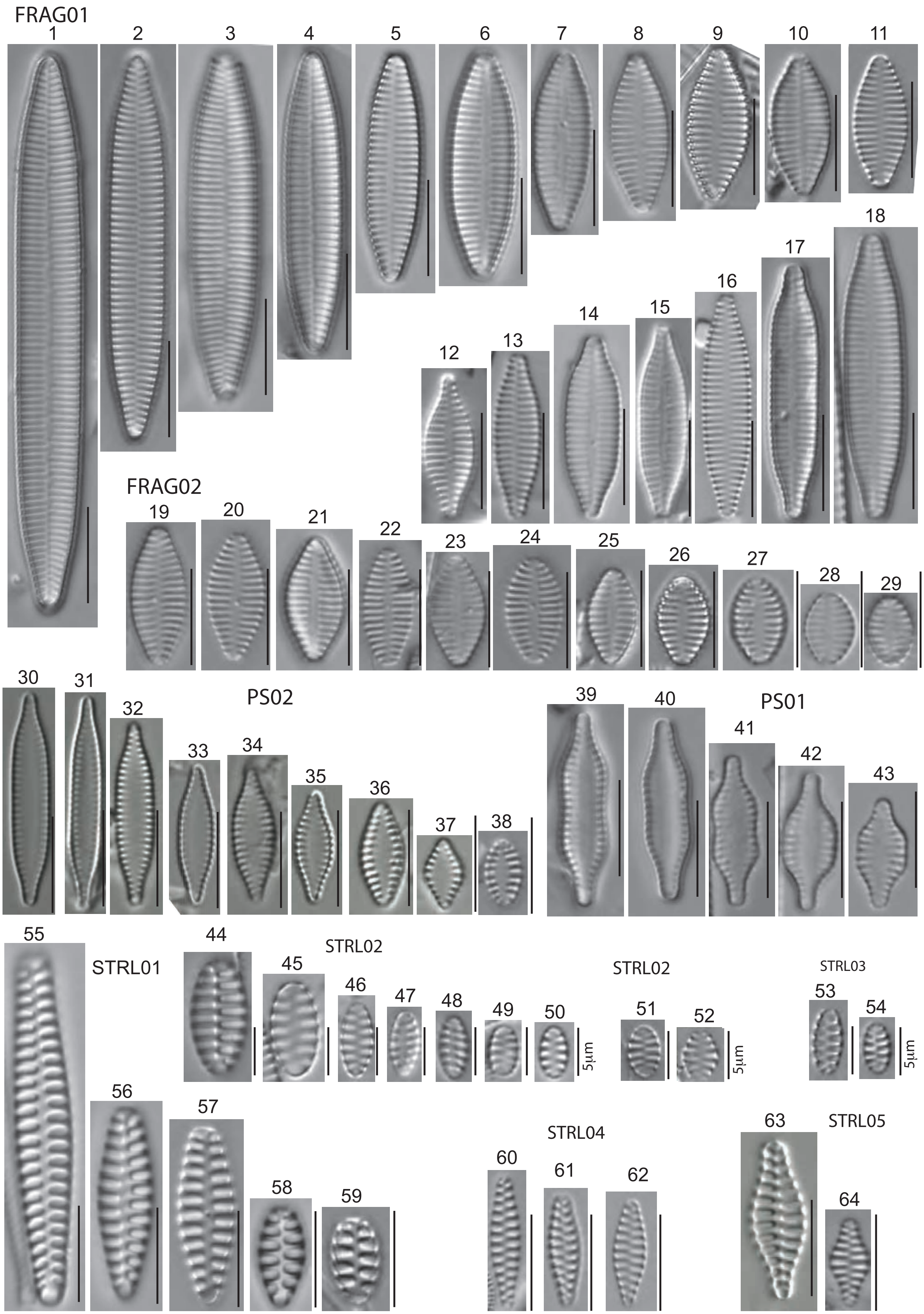

Plate 4 Gomphonemoids

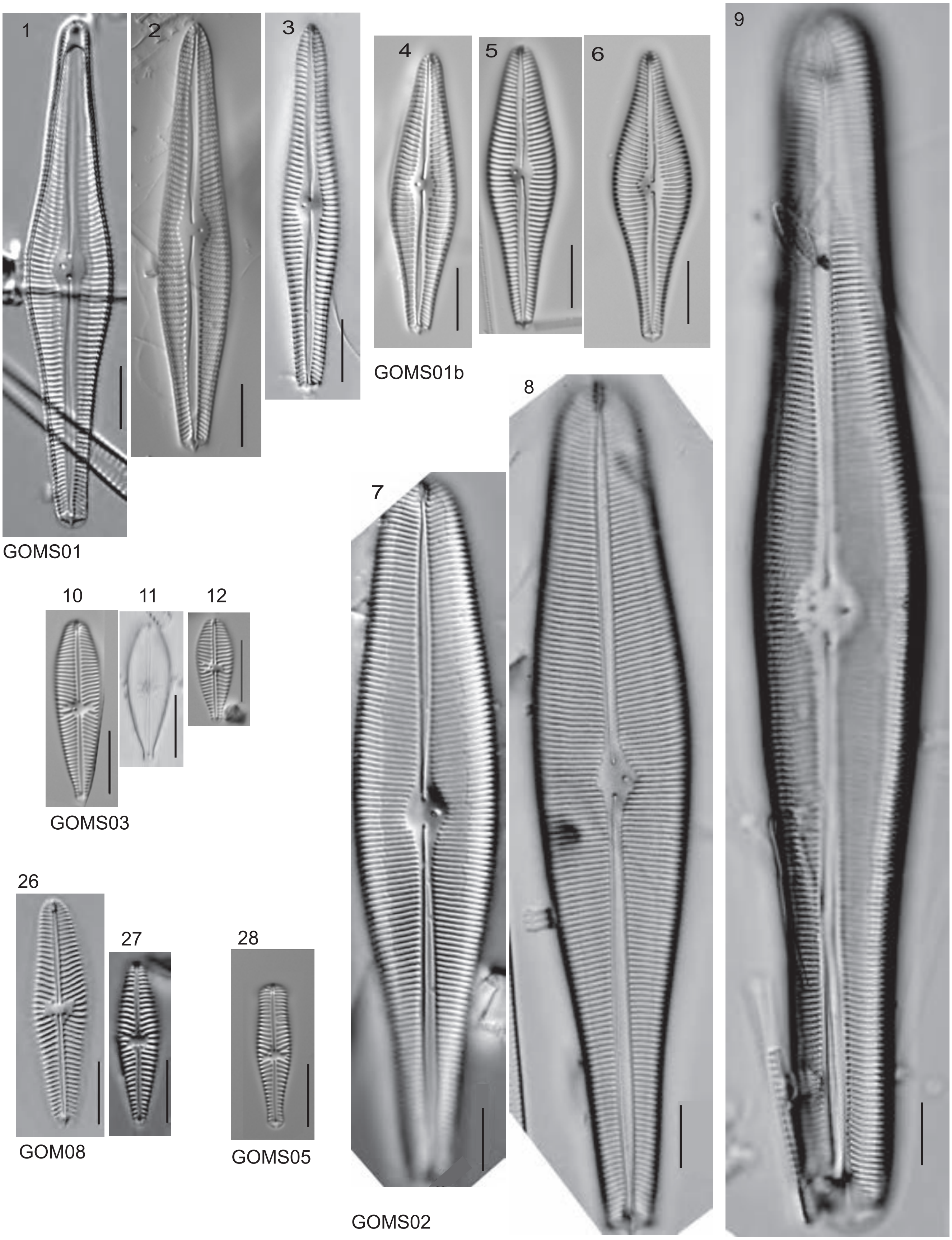

Plate 5 Gomphonemoids

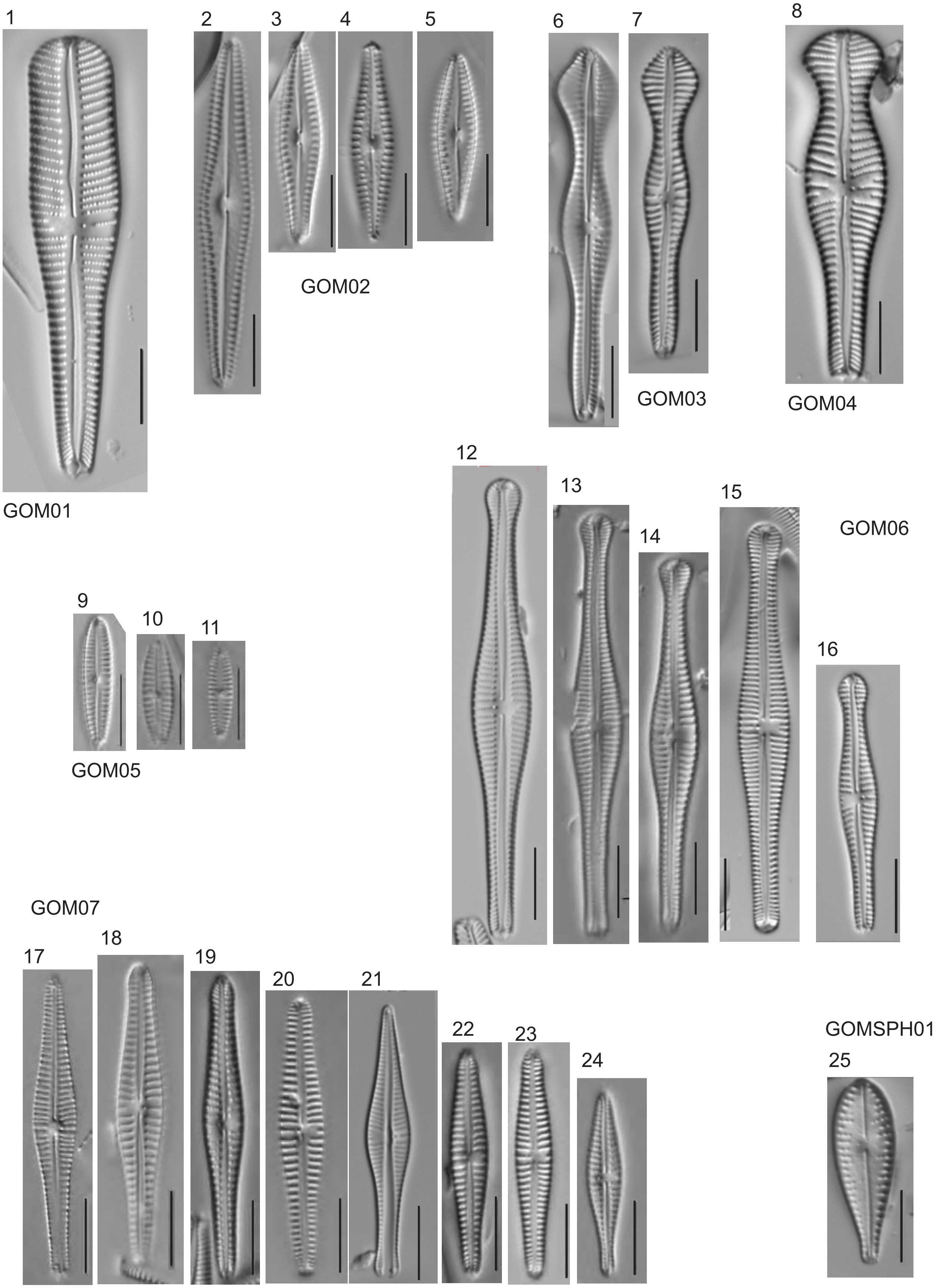

Plate 6 Cymbelloids

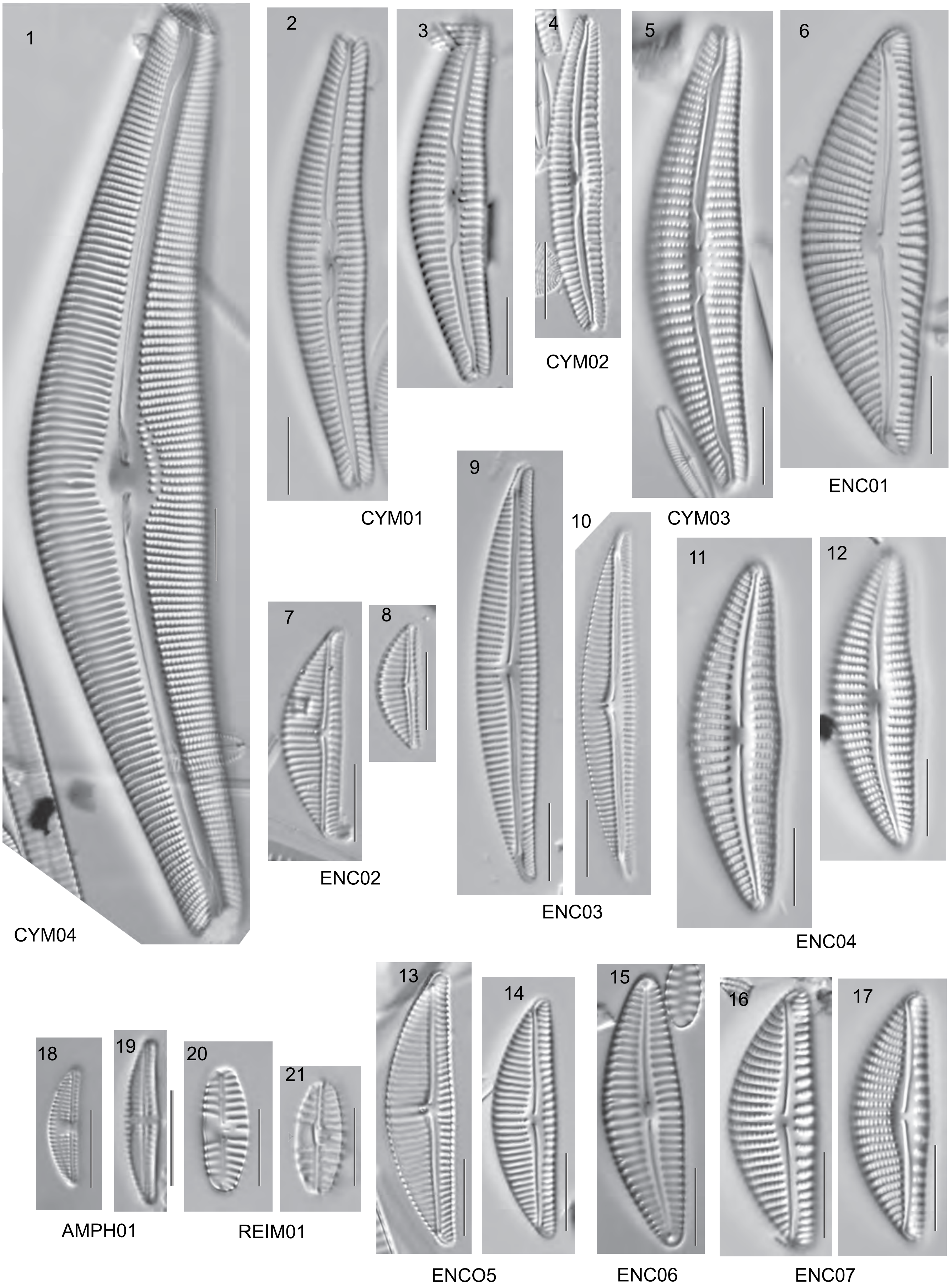

Plate 7 Small biraphids

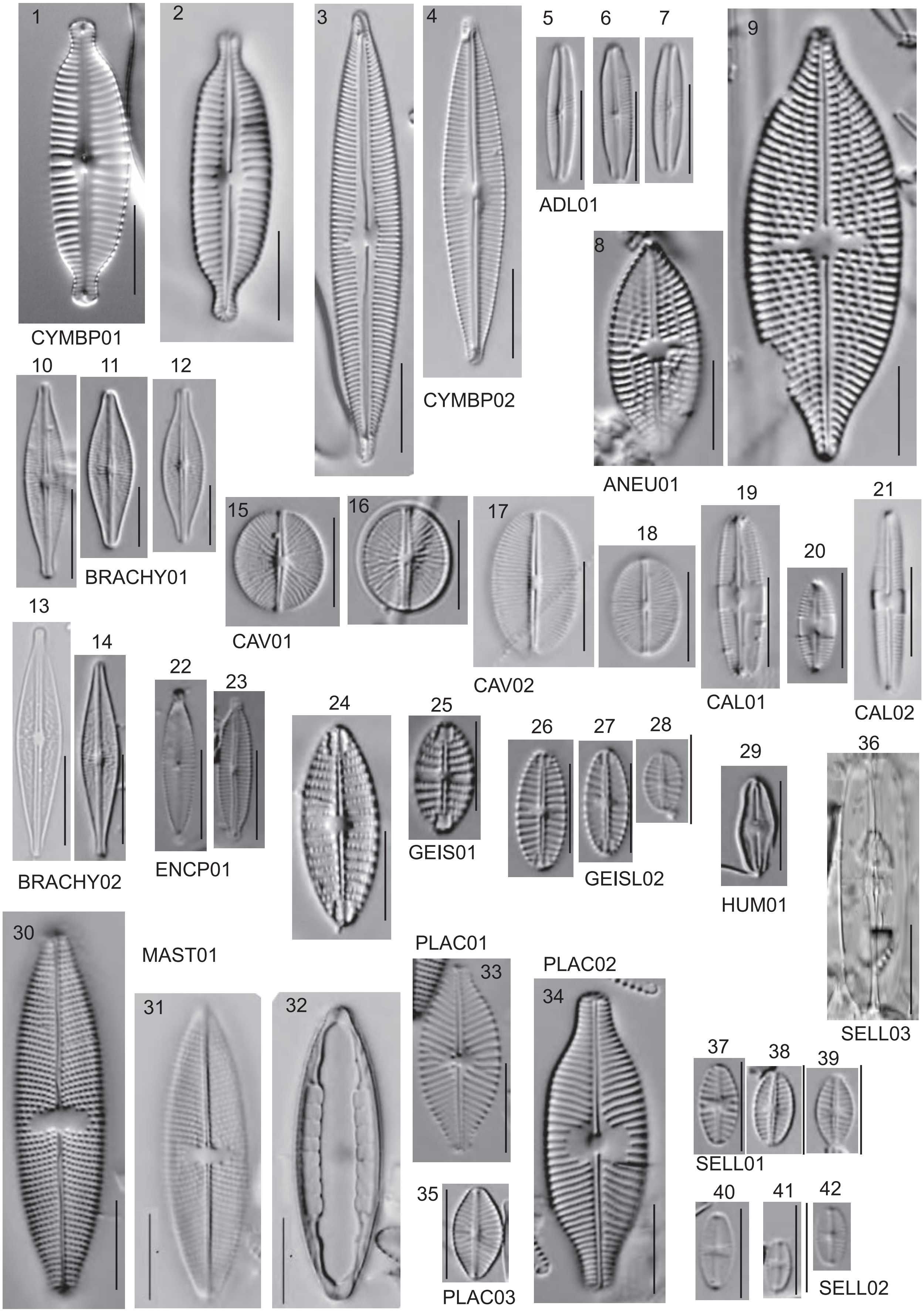

Plate 8 Epithemioids

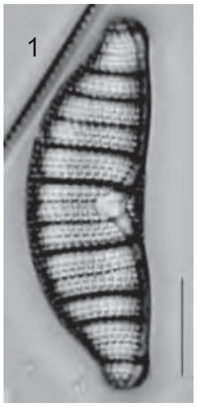

EP01

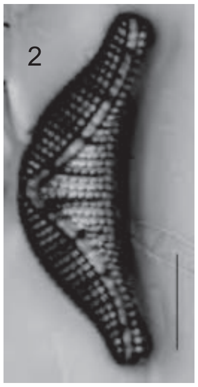

EP02

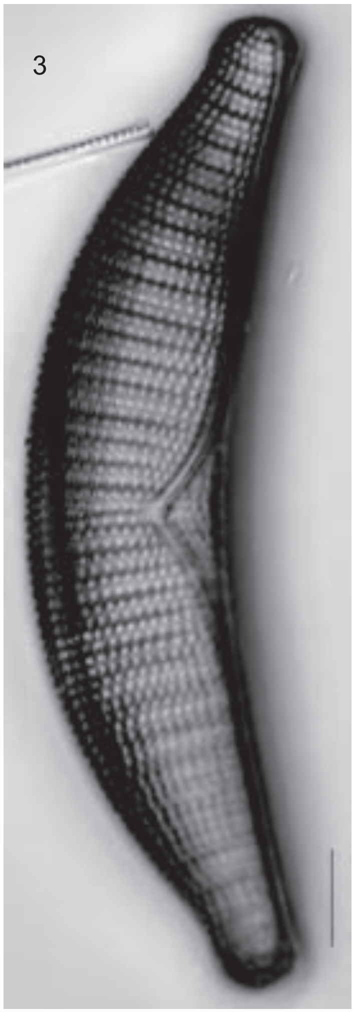

EP03

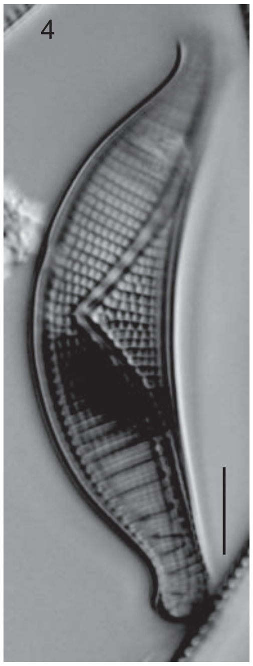

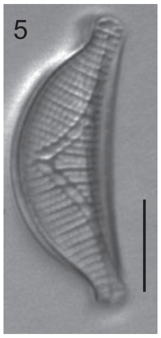

EP04

6

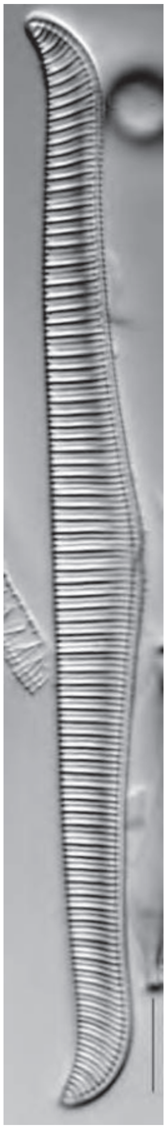

EP05

7

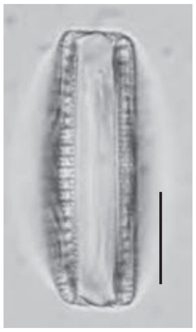

8

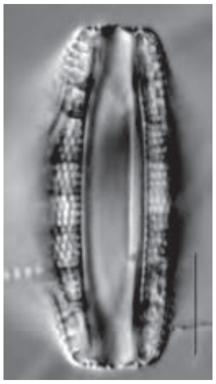

9

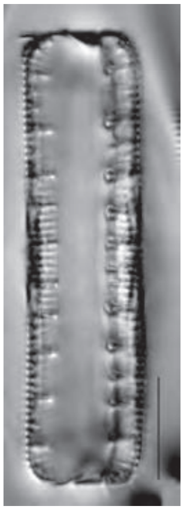

Epithemia girdle views

Plate 9 *Navicula sensu strictu*

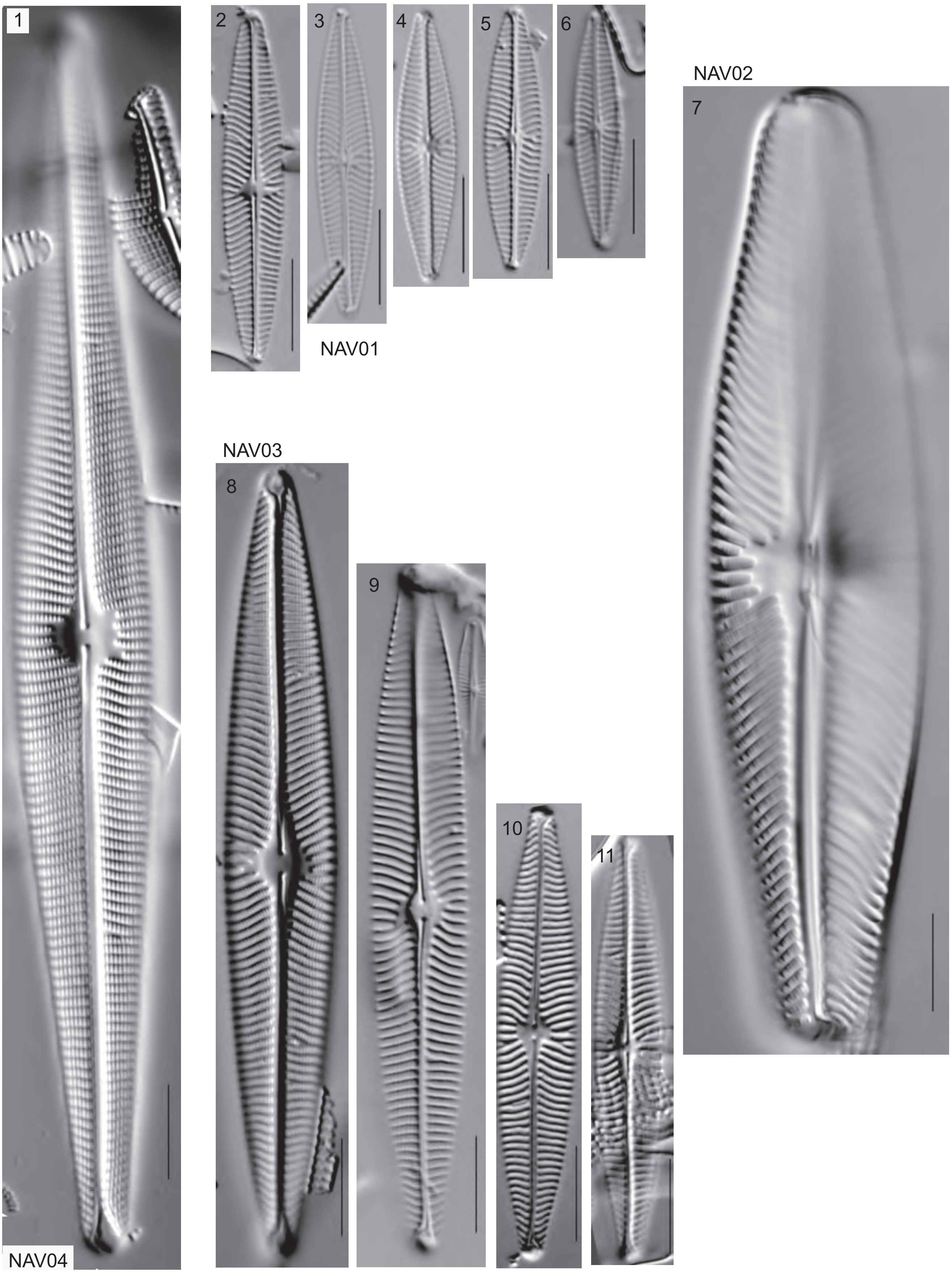

Plate 10 monoraphids

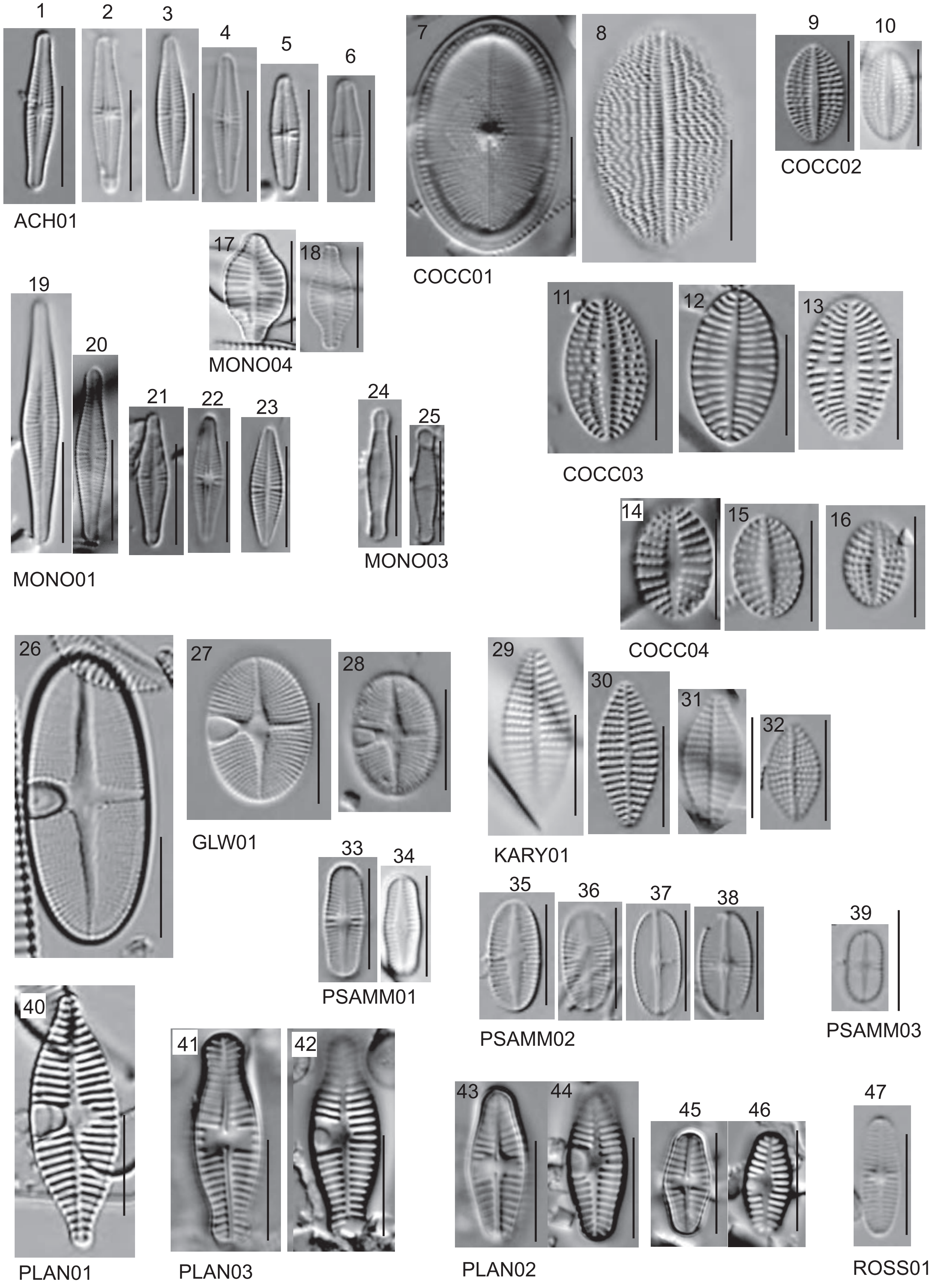

Plate 11 Nitzschioids

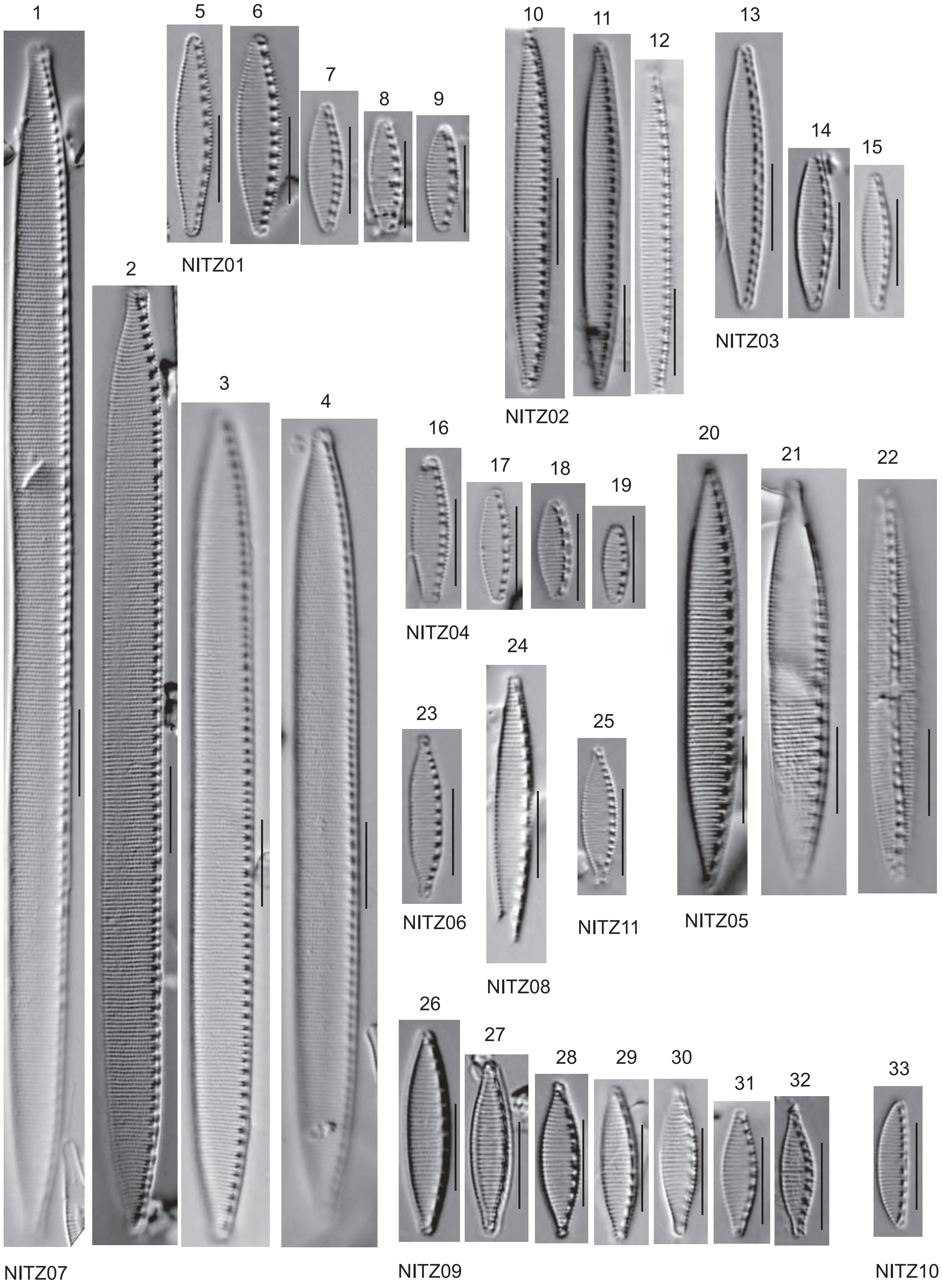

Supplement: Supplement1 [file NIHMS1947911-supplement-Supplement1.zip › Supplementary Data sheet 2.pdf]
