# Supplementary material for: Characterization of algal community composition and structure from the nearshore environment, Lake Tahoe (United States)
Source: Front Ecol Evol. Author manuscript; Available in PMC 2024 Jan 20. (PMC10750852; doi:10.3389/fevo.2022.1053499)
Supplement: Supplement1 [file NIHMS1947911-supplement-Supplement1.zip › Supplementary Data sheet 3.docx]

Diatom Voucher Species references:

Alexson, E.E., Wellard Kelly, H.A., Estepp, L.R., Reavie, E.D. (2018) Morphological variation around the Pantoscekiella comensis complex in the Laurentian Great Lakes, *Diatom Research* 33(3):321-337. <https://doi.org/10.1080/0269249X.2018.1544592>

Antoniades, D., Hamilton, P.B, Douglas, M. S. V., and J.P. Smol. 2008. Diatoms of North America: The freshwater floras of Prince Patrick, Ellef Ringnes and northern Ellesmere Islands from the Canadian Arctic Archipelago. Annotated Diatom Micrographs, vol 17. Ed. Horst Lange-Bertalot. A.R.G. Gantner Verlag K.G. 649 pp.

Bahls, L. (2011). *Navicula aurora*. In Diatoms of North America. Retrieved September 06, 2021, from <https://diatoms.org/species/navicula_aurora>

Bahls, L. (2012a). *Cymbopleura amphicephala*. In Diatoms of North America. Retrieved September 23, 2022, from <https://diatoms.org/species/cymbopleura_amphicephala>

Bahls, L. (2012b). *Aneumastus rostratus*. In Diatoms of North America. Retrieved September 23, 2022, from <https://diatoms.org/species/aneumastus_rostratus>

Bahls, L. (2013) *Stephanodiscus alpinus*. In Diatoms of North America. Retrieved September 04, 2020, from <https://diatoms.org/species/stephanodiscus_alpinus1>

Bahls, L. (2014). *Cymbopleura incertiformis*. In Diatoms of North America. Retrieved September 23, 2022, from <https://diatoms.org/species/cymbopleura_incertiformis>

Bahls, L. (2016a) *Cymbella vulgata*. In Diatoms of North America. Retrieved September 06, 2021, from <https://diatoms.org/species/cymbella_vulgata>

Bahls, L. (2016b). *Geissleria cascadensis*. In Diatoms of North America. Retrieved September 23, 2022, from <https://diatoms.org/species/navicula_cascadensis>

Bahls, L. (2021) *Epithemia smithii*. In Diatoms of North America. Retrieved September 06, 2021, from <https://diatoms.org/species/epithemia_smithii>

Bahls, L. & Bishop, I. (2013) *Gomphonema louisiananum*. In Diatoms of North America. Retrieved September 05, 2021, from <https://diatoms.org/species/gomphonema_louisiananum>

Bishop, I. (2017) *Gomphonema olivaceoides var. densestriata*. In Diatoms of North America. Retrieved September 05, 2020, from <https://diatoms.org/species/gomphonema_olivaceoides_var._denstistriata>

Bishop, I. (2021). *Cocconeis neodiminuta*. In Diatoms of North America. Retrieved September 23, 2022, from <https://diatoms.org/species/cocconeis-neodiminuta>

Burge, D. & Edlund, M. (2015) *Lindavia delicatula*. In Diatoms of North America. Retrieved August 04, 2021, from <https://diatoms.org/species/lindavia_delicatula>

Burge, D. & Edlund, M. (2017a) *Lindavia bodanica*. In Diatoms of North America. Retrieved September 04, 2019, from <https://diatoms.org/species/lindavia_bodanica>

Burge, D., Edlund, M. (2017b). *Placoneis explanata*. In Diatoms of North America. Retrieved September 22, 2022, from <https://diatoms.org/species/placoneis_explanata>

Burge, D., Edlund, M., Manoylov, K., Ognjanova-Rumenova, N., & Hamilton, P. (2016) *Lindavia ocellata*. In Diatoms of North America. Retrieved September 04, 2020, from <https://diatoms.org/species/lindavia_ocellata>

Bryłka, K. & Lee, S. (2019) *Ulnaria capitata*. In Diatoms of North America. Retrieved August 05, 2021, from <https://diatoms.org/species/ulnaria-capitata>

Cantonati, M., Kelly, M.G., Lange-Bertalot, H., *Freshwater benthic diatoms of Central Europe: over 800 common species used in ecological assessment*. Vol. 942. Schmitten-Oberreifenberg: Koeltz Botanical Books, 2017.

Carruthers, W. (1864) The Diatomaceae In: Handbook of British Freshwater Weeds or Algae (J.E. Gray, ed.), London : 75-116.

García,M.L., Echazú, D.M., Romero, O.E., & Maidana , N.I. (2018) *Cocconeis neuquina* Frenguelli (Bacillariophyta): emended description, lectotypification, ecology, and geographical distribution, *Diatom Research* 33(2):219-228. <https://doi.org/10.1080/0269249X.2018.1485596>

Haworth (1990) Diatom name validation, *Diatom Research* 5(1):195-196. <http://dx.doi.org/10.1080/0269249X.1990.9705104>

Jüttner, I., Gurung, S., Sharma, C., Sharma, S., De Hann, M. & Van de Vijver, B. (2010). Morphology of new taxa in the *Cymbella aspera* and *Cymbella neocistula* groups, *Cymbella yakii* sp. nov., and *Cymbella* cf. *hantzschiana* form Everest National Park, Nepal. *Polish Botanical Journal.* 55(1): 73-92.

# Kalinsky, R.G. (1984) Notes on Louisiana diatoms. III. Some new, rare, and interesting diatoms from northwestern Louisiana. In: Proceedings of the Seventh International Diatom Symposium (D.G. Mann, ed.), Otto Koeltz Science Publishers, Koenigstein, 299-306.

Kamae, B. (2015). *Diploneis elliptica*. In Diatoms of North America. Retrieved September 23, 2022, from <https://diatoms.org/species/diploneis_elliptica>

# Kistenich, S., Dreßler, M., Zimmermann, J., Hübener,T., Bastrop, R., & Jahn, R. (2014) An investigation into the morphology and genetics of *Cyclotella comensis* and closely related taxa, *Diatom Research*, 29(4):423-440. <https://doi.org/10.1080/0269249X.2014.922125>

Kociolek, P. (2010) *Hannaea arcus*. In Diatoms of North America. Retrieved August 10, 2021, from <https://diatoms.org/species/hannaea_arcus>

Kociolek, P. (2011a) *Meridion circulare*. In Diatoms of North America. Retrieved August 08, 2021, from <https://diatoms.org/species/meridion_circulare>

Kociolek, P. (2011b) *Gomphoneis pseudo-okunoi*. In Diatoms of North America. Retrieved August 08, 2020, from <https://diatoms.org/species/gomphoneis_pseudookunoi>

Kociolek, P. (2011c) *Gomphonema pusillum*. In Diatoms of North America. Retrieved September 05, 2021, from <https://diatoms.org/species/gomphonema_pusillum>

Kociolek, P. (2011d) *Gomphonema truncatum*. In Diatoms of North America. Retrieved September 05, 2021, from https://diatoms.org/species/gomphonema_truncatum

Kociolek, P. (2011e) Gomphonema kobayasii. In Diatoms of North America. Retrieved September 05, 2021, from <https://diatoms.org/species/gomphonema_kobayasii>

Kociolek, P. (2011f) *Gomphosphenia grovei*. In Diatoms of North America. Retrieved September 05, 2021, from <https://diatoms.org/species/gomphosphenia_grovei>

Kociolek, P. (2011g). *Encyonopsis subminuta*. In Diatoms of North America. Retrieved September 06, 2021, from <https://diatoms.org/species/encyonopsis_subminuta>

Kociolek, P. (2011h). *Mastogloia lacustris*. In Diatoms of North America. Retrieved September 06, 2021, from <https://diatoms.org/species/mastogloia_smithii_var._lacustris>

Kociolek, P. (2011i). *Sellaphora bacillum*. In Diatoms of North America. Retrieved September 06, 2021, from <https://diatoms.org/species/sellaphora_bacillum>

Kociolek, P. (2011j). *Epithemia gibba*. In Diatoms of North America. Retrieved September 06, 2021, from <https://diatoms.org/species/epithemia_gibba>

Kociolek, P. (2011k). *Nitzschia perminuta*. In Diatoms of North America. Retrieved September 06, 2021, from <https://diatoms.org/species/nitzschia_perminuta>

Kociolek, P. (2015) *Gomphonema pygmaeum*. In Diatoms of North America. Retrieved September 05, 2021, from https://diatoms.org/species/gomphonema_pygmaeum

Kociolek, J. P., Kulikovskiy, M.S. & Solak, C.N. (2013) The diatom genus *Gomphoneis* Cleve (Bacillariophyceae) from Lake Baikal, Russia. *Phytotaxa* 154(1): 1-37. http://dx.doi.org/10.11646/phytotaxa.154.1.1

Kociolek, J.P. and Stoermer, E.F. (1988) Taxonomy, Ultrastructure and Distribution of *Gomphoneis herculeana*, *G. eriense* and Closely Related Species (Naviculales: Gomphonemataceae), Proceedings of the Academy of Natural Sciences of Philadelphia, Vol. 140, No. 2(1988), pp. 24-97.

# Kociolek, J.P. and Stoermer, E.F. (1991) Taxonomy and ultrastructure of some Gomphonema

# and Gomphoneis taxa from the Upper Laurentian Great Lakes Canadian Journal of Botany 69: 1557-1576.

# Kociolek, J.P., You, Q.M., Wang, Q.X. & Liu, Q. (2015) A consideration of some interesting freshwater gomphonemoid diatoms from North America and China, and the description of Gomphosinica gen. nov. Nova Hedwigia Supplement 144: 175-198.

Krammer, K. (2002). Diatoms of Europe, Volume 3: Diatoms of The European Inland Waters and Comparable Habitats. *AR G Gantner Verlag K. G Struttgart, Germany*, 515 pp.

# Krammer, K. and Lange-Bertalot, H. (1988) Bacillariophyceae. 2. Teil: Bacillariaceae, Epithemiaceae, Surirellaceae In: Ettl, H., J. Gerloff, H. Heynig and D. Mollenhauer (eds.) Susswasserflora von Mitteleuropa, Band 2/2. Gustav Fisher Verlag, Jena.

# Krammer, K. and Lange-Bertalot H. 2000. Bacillariophyceae 5. English and French translation of the keys. In: Büdel, B., Gärtner, G., Krienitz, L. and Lokhorst, G. M. (eds.). Süßwasserflora von Mitteleuropa. 2(5), 311 pp. Spektrum Akademischer Verlag, Heidelberg-Berlin.

Kulikovskiy, M., Lange-Bertalot, H. and Witkowski, A. (2013) Gliwiczia gen. nov. a new monoraphid diatom genus from Lake Baikal with a description of four species new for science Phytotaxa 109 (1): 1–16. <http://dx.doi.org/10.11646/phytotaxa.109.1.1>

Lange-Bertalot, H., and Ulrich, S., (2014) Contributions to the taxonomy of needle-shaped *Fragilaria* and *Ulnaria* species, *Lauterbornia* 78:1-73.

Levkov, Z., Mitic-Kopanja, D. & Reichardt, E. (2016) The diatom genus *Gomphonema* in the Republic of Macedonia. In: *Diatoms of Europe. Diatoms of the European inland waters and comparable habitats. Volume 8*. (Lange-Bertalot, H. Eds), Oberreifenberg: Koeltz Botanical Books. 552 pp.

Lowe, R. (2010a). *Epithemia adnata*. In Diatoms of North America. Retrieved September 06, 2021, from <https://diatoms.org/species/epithemia_adnata>

Lowe, R. (2010b). *Epithemia sorex*. In Diatoms of North America. Retrieved September 23, 2022, from <https://diatoms.org/species/epithemia_sorex>

Lowe, R. (2015) *Discostella stelligera*. In Diatoms of North America. Retrieved September 04, 2019, from <https://diatoms.org/species/discostella_stelligera>

Metzeltin, D. & Lange-Bertalot, H. (1998) Tropical diatoms of South America I: About 700 predominantly rarely known or new taxa representative of the neotropical flora. In: Lange-Bertalot, H. (ed.), Iconographia Diatomologica. Annotated Diatom Micrographs. Diversity-Taxonomy-Geobotany. Koeltz Scientific Books. Königstein, Germany, 5:695 pp.

Morales E.A., (2003) On the taxonomic status of the genera *Belonastrum* and *Synedrella* proposed by Round and Maidana (2001). Cryptogamie Algol.24(3):277-288.

Morales, E.A. (2005) Observations of the morphology of some known and new fragilarioid diatoms (Bacillariophyceae) from rivers in the USA. *Phycological Research* 53(2): 113-133.

Morales, E. A.(2010a) *Fragilaria vaucheriae*. In Diatoms of North America. Retrieved August 08, 2021, from <https://diatoms.org/species/fragilaria_vaucheriae>

Morales, E. (2010b) *Staurosirella pinnata.* In Diatoms of North America. Retrieved August 10, 2021, from <https://diatoms.org/species/staurosirella_pinnata>

Morales, E. (2010c) *Pseudostaurosira brevistriata*. In Diatoms of North America. Retrieved August 09, 2021, from <https://diatoms.org/species/pseudostaurosira_brevistriata>

Morales, E. (2010d) *Staurosirella leptostauron var. dubia*. In Diatoms of North America. Retrieved August 10, 2021, from <https://diatoms.org/species/staurosirella_leptostauron_var._dubia>

Morales, E. (2011) *Pseudostaurosira elliptica*. In Diatoms of North America. Retrieved August 10, 2021, from <https://diatoms.org/species/pseudostaurosira_elliptica>

Morales, E.A. & Edlund, M.B. (2003) Studies in selected fragilarioid diatoms (Bacillariophyceae) from Lake Hovsgol, Mongolia. Phycological Research 51(4): 225-239.

Morales, E. & Rosen, B. (2013) *Fragilaria tenera*. In Diatoms of North America. Retrieved August 08, 2021, from <https://diatoms.org/species/fragiaria_tenera>

Otu, M., Spaulding, S. (2011). *Cavinula pseudoscutiformis*. In Diatoms of North America. Retrieved September 06, 2021, from <https://diatoms.org/species/cavinula_pseudoscutiformis>

Patrick, R.M. and Reimer, C.W. (1966) The Diatoms of the United States exclusive of Alaska and Hawaii, V. 1 Monographs of the Academy of Natural Sciences of Philadelphia 13

# Patrick, R.M. and Reimer, C.W. (1975) The Diatoms of the United States, exclusive of Alaska and Hawaii, V. 2 Monographs of the Academy of Natural Sciences of Philadelphia 13

Pedraza Garzon, E. (2014) *Staurosirella martyi*. In Diatoms of North America. Retrieved September 04, 2019, from <https://diatoms.org/species/staurosirella_martyi>

Pickett, F., Spaulding, S. (2011). *Gomphosinica geitleri*. In Diatoms of North America. Retrieved September 06, 2021, from <https://diatoms.org/species/gomphosinica_geitleri>

Polaskey, M. (2018a). *Cavinula jaernefeltii*. In Diatoms of North America. Retrieved September 23, 2022, from <https://diatoms.org/species/cavinula-jaernefeltii>

Polaskey, M. (2018b). *Humidophila perpusilla*. In Diatoms of North America. Retrieved September 23, 2022, from <https://diatoms.org/species/humidophila-perpusilla>

Polaskey, M. (2019). *Adlafia suchlandtii*. In Diatoms of North America. Retrieved September 06, 2021, from <https://diatoms.org/species/adlafia-suchlandtii>

Potapova, M. (2009a) *Odontidium mesodon*. In Diatoms of North America. Retrieved August 08, 2021, from <https://diatoms.org/species/odontidium_mesodon>

Potapova, M. (2009b) *Reimeria sinuata*. In Diatoms of North America. Retrieved September 06, 2021, from <https://diatoms.org/species/reimeria_sinuata>

Potapova, M. (2009c). *Geissleria acceptata*. In Diatoms of North America. Retrieved September 06, 2021, from <https://diatoms.org/species/geissleria_acceptata>

Potapova, M. (2009d). *Achnanthidium minutissimum*. In Diatoms of North America. Retrieved September 06, 2021, from <https://diatoms.org/species/achnanthidium_minutissimum>

Potapova, M. (2009e). *Rossithidium pusillum*. In Diatoms of North America. Retrieved September 06, 2021, from <https://diatoms.org/species/rossithidium_pusillum>

Potapova, M. (2010a) *Aulacoseira pusilla*. In Diatoms of North America. Retrieved September 04, 2019, from <https://diatoms.org/species/aulacoseira_pusilla>

Potapova, M. (2010b). *Karayevia clevei*. In Diatoms of North America. Retrieved September 23, 2022, from <https://diatoms.org/species/karayevia_clevei>

Potapova, M. (2010c). *Psammothidium levanderi*. In Diatoms of North America. Retrieved September 06, 2021, from <https://diatoms.org/species/psammothidium_levanderi>

Potapova, M. (2010d). *Psammothidium didymum*. In Diatoms of North America. Retrieved September 06, 2021, from <https://diatoms.org/species/psammothidium_didymum>

Potapova, M. (2010e). *Planothidium apiculatum*. In Diatoms of North America. Retrieved September 06, 2021, from https://diatoms.org/species/planothidium_apiculatum

Potapova, M. (2010f). *Planothidium frequentissimum*. In Diatoms of North America. Retrieved September 04, 2019, from <https://diatoms.org/species/planothidium_frequentissimum>

Potapova, M. (2011a). *Navicula radiosa*. In Diatoms of North America. Retrieved September 06, 2021, from <https://diatoms.org/species/navicula_radiosa>

Potapova, M. (2011b). *Gliwiczia calcar*. In Diatoms of North America. Retrieved September 23, 2022, from <https://diatoms.org/species/gliwiczia_calcar>

Potapova, M., Spaulding, S. (2013). Cocconeis placentula sensu lato. In Diatoms of North America. Retrieved September 22, 2022, from <https://diatoms.org/species/cocconeis_placentula>

# Reichardt, E. and Lange-Bertalot, H. (1991) Taxonomische Revision des Artencomplexes um Gomphonema angustum—G. dichotomum—G. intricatum—G. vibrio und ahnliche Taxa (Bacillariophyceae). Nova Hedwigia 53(3-4): 519-544.

# Reichardt, E. (2001) Revision der Arten um Gomphonema truncatum und G. capitatum In Jahn R., Kociolek, J.P., Witkowski, A. and Compère, P. (Eds.). Lange-Bertalot-Festschrift, Studies on Diatoms. A.R.G. Gantnter Verlag K.G. Ruggell. p. 187-224

Reichardt, E. (2015) Taxonomy and distribution of Gomphonema subtile Ehrenberg (Bacillariophyceae and six related taxa, *Fottea Olomouc*, 15(1):27-38.

Sala, S.E., Guerrero, J.M. & Ferrario, M.E. (1993) Redefinition of Reimeria sinuata (Gregory) Kociolek & Stoermer and recognition of Reimeria uniseriata nov. spec. Diatom Research 8(2):439-446.

Scheffler,W. Houk,V, & Klee, R (2003) Morphology, morphological variability and ultrastructure of *Cyclotella delicatula* Hustedt (Bacillariophyceae) from Hustedt material, *Diatom Research* 18(1):107-121, <https://doi.org/10.1080/0269249X.2003.9705576>

Schulte, N. (2014). *Fragilaria synegrotesca*. In Diatoms of North America. Retrieved September 06, 2021, from https://diatoms.org/species/fragilaria_synegrotesca

# Sovereign, H.E. (1958) The diatoms of Crater Lake, Oregon Transactions of the American Microscopical Society 77(2):96-134. <https://doi.org/10.2307/3224112>

Spaulding, S. (2010). *Encyonema reimeri*. In Diatoms of North America. Retrieved September 23, 2022, from <https://diatoms.org/species/encyononema_reimeri>

Spaulding, S., Bishop, I. (2014) *Encyonema pergracile*. In Diatoms of North America. Retrieved September 06, 2021, from <https://diatoms.org/species/encyonema_pergracile>

Stepanek, J., Kociolek, P. (2011). *Amphora pediculus*. In Diatoms of North America. Retrieved September 23, 2022, from <https://diatoms.org/species/amphora_pediculus>

Stratton, L.E., (2013) Feasibility study for using diatom assemblages from a small dilute subalpine lake as an indicator of past megadroughts in the Sierra Nevada, MS Thesis University of Nevada Reno, 239 pp. <http://hdl.handle.net/11714/3180>

TERC, Tahoe Environmental Research Center (2011) Tahoe: state of the lake reports (14 annual volumes). Univ Calif Davis.

Tuji, A. (2009) Examination of Type Material and typification of seven diatoms described C.G. Ehrenberg. In: Tanumura & Aita, Y. (Eds.): Joint Haeckel and Ehrenberg Project: Reexamination of the Haeckel and Ehrenberg Microfossil Collections as a Historical and Scientific Legacy. *Natural Museum of Nature and Science Monographs*, Tokyo 40: 13-21.

Walls, J. (2016) *Gomphonema sarcophagus*. In Diatoms of North America. Retrieved September 05, 2021, from <https://diatoms.org/species/gomphonema_sarcophagus>

# Wetzel, C.E., Ector, L., Van de Vijver, B., Compère, P. and Mann, D.G. (2015) Morphology, typification and critical analysis of some ecologically important small naviculoid species (Bacillariophyta) Fottea, Olomouc 15(2): 203–234.

Wetzel, C.E., Van de Vijver, B., Kopalová, K., Hoffmann, L., Pfister, L. and Ector, L. (2014) Type analysis of the South American diatom *Achnanthes haynaldii* (Bacillariophyta) and description of *Planothidium amphibium* sp. nov., from aerial and aquatic environments in Oregon (USA) Plant Ecology and Evolution 147 (3): 439-454. <https://doi.org/10.5091/plecevo.2014.1058>

White, C. (2011). *Cymbella neocistula*. In Diatoms of North America. Retrieved September 23, 2022, from <https://diatoms.org/species/cymbella_neocistula>

Wojtal, A. Z., Ector, L., Van de Vijver, B., Morales, E. A., Blanco, S., Piatek, J., & Smieja, A. (2011). *The Achnanthidium minutissimum* complex (Bacillariophyceae) in southern Poland. Algological Studies, 211-238. <https://doi.org/10.1127/1864-1318/2011/0136-0211>

Soft-bodied algae taxonomic references:

Komárek, J. (2013). Cyanoprokaryota. 3. Heterocytous Genera. Süsswasser flora von Mitteleuropa, 19/3. Springer, Heidelberg.

Komárek, J., and Anagnostidis, K. (1986). Modern approach to the classification system of cyanophytes. 2. Chroococcales. *Algol Stud.* 43, 157–226.

Komárek, J., and Anagnostidis, K. (1989). Modern approach to the classification system of cyanophytes. 4 – Nostocales. *Algological Studies/Archiv fur Hydrobiologie*, Supplement Volumes 56, 247–345.

Komárek, J., and Anagnostidis, K. (1998). Cyanoprokaryota I: Chroococcales, Süsswasser flora von Mitteleuropa, 19/1. G. Fischer, Stuttgart.

Komárek, J., and Anagnostidis, K. (2005). Cyanoprokaryota II: Oscillatoriales, Süsswasser flora von Mitteleuropa, 19/2. G. Fischer, Stuttgart.

Komárek, J, and Hauer T. (2015). CyanoDB.cz—On-line database of cyanobacterial genera. Wordwide electronic publication, Univ. of South Bohemia and Inst. of Botany. http://www.cyanodb.cz . Accessed: 5 Aug. 2021.

Komárek, J., and Johansen J. (2015a). Coccoid cyanobacteria. In: Wehr JD, Sheath RG, Kociolek JP (eds). Freshwater algae of North America. Ecology and classification. Academic, San Diego, pp 75–133.

Komárek J, and Johansen J (2015b). Filamentous cyanobacteria. In: Wehr JD, Sheath RG, Kociolek JP (eds) Freshwater algae of North America. Ecology and classification. Academic, San Diego, pp. 135–235.

Komárek , J., Jezberov J., Komárek, O. et al (2010). Variability of Chroococcus (Cyanobacteria) morphospecies with regard to phylogenetic relationships. *Hydrobiologia* 639, 69–83.

Prescott, G. W. (1931). Iowa Algae. University of Iowa, Iowa City, Iowa, 235 pp.

Prescott, G. W. (1962). The Algae of the Western Great Lakes Area. Wm. C. Brown Co., Dubuque, Iowa, 977 pp.

Stancheva, R., Hall, J. D., McCourt, R. M. and Sheath, R. J. (2013). Identity and phylogenetic placement of Spirogyra species (Zygnematophyceae, Charophyta) from California streams and elsewhere. *J. Phycol*. 49, 588–607.

Stancheva, R., Fuller, C., and Sheath, R. G. (2014). Soft-Bodied Stream Algae of California. <http://dbmuseblade.colorado.edu/DiatomTwo/sbsac_site/index.php>

Starmach, K. (1966). Cyanophyta-Sinice Glaucophyta-Glaukofity. Flora Slodkowodna Polski. Panstowowe Wydawnictwo Naukowe. Vol 2.

Wehr, J. D., Sheath R. G., and Kociolek J. P. (2012). Freshwater algae of North America. Ecology and Classification. Aquatic Ecology Series. Academic, San Diego.
